# LASSO-type instrumental variable selection methods with an application to Mendelian randomization

**DOI:** 10.1177/09622802241281035

**Published:** 2024-11-15

**Authors:** Muhammad Qasim, Kristofer Månsson, Narayanaswamy Balakrishnan

**Affiliations:** 1 Jönköping International Business School, 4161 Jönköping University, Jönköping, Sweden; 2 Department of Mathematics and Statistics, McMaster University, Hamilton, ON, Canada

**Keywords:** Causal inference, instrumental variable, model selection, LASSO, jackknife, heteroscedasticity, C13, C26, C36

## Abstract

Valid instrumental variables (IVs) must not directly impact the outcome variable and must also be uncorrelated with nonmeasured variables. However, in practice, IVs are likely to be invalid. The existing methods can lead to large bias relative to standard errors in situations with many weak and invalid instruments. In this paper, we derive a LASSO procedure for the *k*-class IV estimation methods in the linear IV model. In addition, we propose the jackknife IV method by using LASSO to address the problem of many weak invalid instruments in the case of heteroscedastic data. The proposed methods are robust for estimating causal effects in the presence of many invalid and valid instruments, with theoretical assurances of their execution. In addition, two-step numerical algorithms are developed for the estimation of causal effects. The performance of the proposed estimators is demonstrated via Monte Carlo simulations as well as an empirical application. We use Mendelian randomization as an application, wherein we estimate the causal effect of body mass index on the health-related quality of life index using single nucleotide polymorphisms as instruments for body mass index.

## 1. Introduction

The instrumental variable (IV) technique is one of the most commonly used causal inference methods for analyzing observational and experimental studies with unmeasured confounders. This technique is based on three important assumptions.^1^ The first assumption is relevance, which requires that the exposure not be independent of the instrument. The second assumption is exclusion, which requires the instrument's impact on the outcome to be completely mediated by the exposure. The final assumption is the independence of confounding factors (unmeasured variables). An example of IV analysis in medical statistics is Mendelian randomization (MR), wherein genetic data are used as instruments to distinguish causation from correlation while analyzing the effects of adjustable risk factors (e.g. body mass index, blood pressure, and alcohol intake) on health, social and economic outcomes. However, a difficult task in MR is identifying IVs that fulfill the above-stated assumptions.^2^

One challenge regarding the relevance assumption is when instruments (e.g. genetic markers) are only weakly associated with the outcome variable. Staiger and Stock^3^ derived the effects of weak instruments on the linear IV model, which led to the development of a simple *F*-test for weak instruments introduced by Stock and Yogo.^4^ Seng and Li^5^ proposed a model averaging method to address the issue of high-dimensional and weak instruments. Qasim et al.^6^ suggested weighted average *K*-class IV methods to address the issue of many weak instruments. However, these methods are developed under the assumption that all the instruments are valid. A second challenge is potential heteroscedasticity, which can bias the classical two-stage least squares (TSLS) estimator, as demonstrated by Angrist et al.^7^ A third challenge arises when some available instruments are invalid, as they may directly affect the outcome of interest. If IVs are uncorrelated, this issue can be addressed via methods from the meta-analysis literature. When all instruments are valid, the inverse-variance weighted method can be employed, and if a majority of the instruments are valid, then the median estimator, as suggested by Bowden et al.,^8^ can be used. Further enhancements to these estimators are described in Burgess et al.^9^ In recent work, Seng et al.^10^ used model averaging in the linear IV model to address the challenge of high dimensionality. This model averaging approach uses different subsets of single nucleotide polymorphisms (SNPs) as instruments to predict exposure, followed by weighting the submodel predictions via penalization methods.

With potentially correlated instruments and if no prior knowledge exists regarding the validity of the instruments, this problem can instead be treated as a model selection problem. This approach is more informative since it also shows which instruments are in fact invalid and have a direct effect on the outcome variable. Andrews^11^ introduced the moment selection criterion (MSC) for the IV model, which is estimated via the generalized method of moments. However, this method becomes computationally infeasible when the number of instruments is large. For this reason, Kang et al.^12^ proposed a LASSO-type procedure for TSLS, which is as computationally fast as ordinary least squares (OLS). Even without prior knowledge of the instrument's validity, this method can identify valid instruments and estimate the causal effect under the weak condition that the proportion of invalid instruments is strictly less than 50% of the total instruments. Windmeijer et al.^13^ further developed this method and introduced the adaptive LASSO (ALASSO) approach, which can be used when invalid instruments are relatively strong. Lin et al.^14^ introduced a robust IV estimation method to overcome the issue of many weak and invalid instruments via a surrogate sparsest penalty. Moreover, accurate causal inference without selecting instruments, especially in the context of Mendelian randomization methods from the meta-analysis literature, has been considered. Notable examples are the median^8^ and mode^15^ estimators. Using the flexible variable selection approach that allows for correlated instruments, we show that one can find robust estimators for both weak instruments and heteroscedasticity.

The first contribution of this paper is that it adds to this growing research field by addressing the issue of invalid instruments under many weak instruments. According to Hernan and Robins^16^ and Davies et al.,^2^ in the presence of weak instruments, even minor deviations from the exclusion assumption cause large bias in the estimated causal effect. Therefore, this is a particularly important empirical situation to examine. By following Kang et al.,^12^ we derive a LASSO procedure for the limited information maximum likelihood (LIML) estimator and FUL^17^ estimator. We primarily consider situations with a single outcome and a single risk factor. Burgess et al.^18^ stated that the methods do not significantly differ in this situation; the main difference is that LIML estimates parameters only from a single equation, whereas FUL uses a three-stage least squares approach and estimates the model simultaneously as a system of equations. When LIML is used, not all moments are defined, but FUL does not suffer from this, as mentioned by Hahn et al.^19^ A significant advantage of LIML and FUL over TSLS is that the median of the distribution of the LIML estimator is close to being unbiased in the presence of many weak instruments.^18^

The second contribution of the paper is the use of the jackknife technique to derive heteroskedasticity-robust versions of the LASSO type of estimators for TSLS, LIML and FUL. Angrist et al.^7^ showed that the TSLS is biased in both situations and suggested a jackknife approach that performs better. Furthermore, Hausman et al.^20^ showed that the LIML estimator is biased and presented some conditions under which it is even inconsistent in the presence of many instruments and heteroscedasticity. These authors then derived heteroskedasticity-robust versions of the LIML and FUL estimators (denoted as HLIML and HFUL, respectively). In this paper, we derive the jackknife version of the sisVIVE^12^ estimator in the presence of many invalid instruments; this estimator is robust to heteroscedasticity. We also derive jackknife versions of the LIML and FUL estimators, which provide comparatively easy solutions to the problem of many invalid and valid instruments in the case of heteroscedastic data. Additionally, for convenience, we created an R package for implementing the proposed methods.^1^

We show in the Monte Carlo simulation study that the LIML and FUL estimators yield substantial improvements in high-dimensional instrumental variable studies. These improvements are especially pronounced for many weak instruments. Our simulation results also reveal substantial improvements in the bias and median square error (MSE) when the jackknife approach is used for both heteroscedastic and homoscedastic data. Therefore, we recommend that researchers and practitioners use the jackknife technique, especially in the presence of heteroscedasticity. In real-life applications, we use all of the suggested estimators in an MR study in which we estimate the causal effect of body mass index (BMI) on the health-related quality of life index (HRQLI) via SNPs as instruments for BMI. Owing to the presence of heteroscedasticity and weak instruments, the jackknife IV method performs the best in this case and yields quite reasonable results.

The remainder of this paper is organized as follows. In Section 2, the model construction and notations used are discussed, and the valid and invalid instruments in the linear IV model are defined. The LASSO-type robust estimation method is introduced, and its properties and theoretical performance are then discussed in Section 3. The simulation study and empirical application are detailed in Sections 4 and 5, respectively. Finally, some concluding remarks are provided in Section 6. All mathematical proofs are provided in Appendix Sections A–C of the supplementary materials.

## 2. Model construction

We define the causal model by following the lines of Kang et al.^12^ and Small.^21^ Suppose we have *n* observations $\left(Y_{i},X_{i},Z_{i.}:i=1,\ldots ,n\right)$ that are independently and identically distributed, where $Y_{i}\in R^{1}$ and $X_{i}\in R^{1}$ represent the observed outcome and the exposure (endogenous) variable, respectively, and the variables $Z_{i}\in R^{L}$ are the IVs. The model for the random sample is given by

(2.1) $$Y_{i}=X_{i}\beta_{0}+Z_{i.}^{T}\delta_{0}+e_{i},\;E(e_{i}|Z_{i.})=0,$$

where $\beta_{0}$ and $\delta_{0}$ are the true parameters, $e_{i}$ is an error term and $\beta_{0}$ is the causal parameter of interest. We further assume that $E[e_{i}^{2}|Z_{i.}]=\sigma_{e}^{2}$ and let $\delta_{0}=\gamma_{0}+\Gamma_{0}$ , where $\gamma_{0}$ represents the direct effect of the IVs on the outcome and where $\Gamma_{0}$ represents the association between the IVs and the confounders. By defining $\hat{\psi }=(Z^{T}Z{)}^{-1}Z^{T}X$ such that $\hat{X}=P_{Z}X$ with the i*th* element of ${\hat{X}}_{i}$ being ${\hat{X}}_{i}=Z_{i.}^{T}\hat{\psi }$ **,** we define

(2.2) $$X_{i}=Z_{i.}^{T}\psi_{0}+\mu_{i},$$

where $\psi_{0}=(E[Z_{i.}^{T}Z_{i.}]{)}^{-1}E[Z_{i.}X_{i}]$ and where $\mu_{i}$ is an error term; therefore, $E[Z_{i.}\mu_{i}]=0$ . Both $e_{i}$ and $\mu_{i}$ are random errors and let $\xi_{i}=(e_{i}\mu_{i}{)}^{T}$ . The mean is $E[\xi_{i}]=0$ , and the variance–covariance matrix is $E[\xi_{i}\xi_{i.}^{T}]=\left[\begin{array}{cc} \sigma_{e}^{2} & \sigma_{e\mu }\\ \sigma_{\mu e} & \sigma_{\mu }^{2} \end{array}\right]$ . In addition, the assumption of the error terms under the setting of homoscedasticity and heteroscedasticity is discussed in Assumption 1.3. Kang et al.^12^ emphasized the uniqueness of the solutions for parameters $\beta_{0}$ and $\delta_{0}$ and discussed necessary and sufficient conditions for identifying $\beta_{0}$ and $\delta_{0}$ . If $\gamma_{0}=0$ , then there is no direct effect of instruments on the outcome, and similarly, if $\Gamma_{0}=0$ , then there are no confounders because $\delta_{0}=0$ . The value of $\delta_{0}$ encompasses the concept of valid and invalid instruments. Therefore, the definition of valid and invalid instruments states that the instruments (j=1,…,L) are valid when $\delta_{0,j}=0$ and that the instruments (j=1,…,L) are invalid when $\delta_{0,j}\neq 0$ . Assume that $Z_{IN}$ is the set of invalid instruments, where $IN=(j=1,\ldots ,L:\delta_{0,j}\neq 0)$ and $\delta_{IN}\in R^{r}$ is the coefficient vector of invalid instruments. The definition of valid instruments corresponds to the formal definition of Holland^22^ and a special case of the valid instrument's definition of Angrist et al.^23^ when L=1 . The theory of valid IVs can be perceived as a simplification of Holland's^22^ model when L>1 . Let r=0,1,…,L−1 denote the number of invalid instruments that are below the upper bound, U=r+1 , i.e. r<U . For any full-rank matrix $Z\in R^{n\times L}$ , $M_{\text{Z}}=I_{n}-P_{\text{Z}}$ is the residual-forming matrix, where $P_{\text{Z}}=Z(Z^{T}Z{)}^{-1}Z^{T}$ is the projection matrix onto the column space of Z and where $I_{n}$ is an identity matrix of n×n . The *l_p_*-norm is denoted by $\|\cdot {\|}_{p}$ so that the $l_{0}\text{ - norm}$ corresponds to $\|\cdot {\|}_{0}$ , which yields the number of nonzero components of a vector, and the $l_{\infty }\text{ - norm}$ is denoted by $\|\cdot {\|}_{\infty }$ , which yields the maximum element of a vector. We have, for example, $\delta_{0}$ , which represents the number of nonzero components in δ **.** The vector δ is known as *r* -sparse if it contains r≤L nonzero elements. Let S⊆(1,2,…L) be any set and let $S^{c}$ denote the complement of set *S*. Furthermore, let $\text{supp}(\delta )=\{j:\delta_{j}\neq 0\}$ denote the support of δ . If $A\in R^{m\times n}$ and $B\in R^{m\times n}$ are two matrices, their inner product is defined as $\{A,B\}=\text{tr}(A^{T}B)=\sum_{i=1}^{m}\sum_{k=1}^{n}a_{ik}b_{ik}$ .

The basic definitions of the restricted isometry (RI) property and restricted orthogonality constant (ROC) are given by Khosravy et al.,^24^ Cai and Zhang^25^ and Cai et al.^26^ We use Definitions 2.1 and 2.2 below to analyze the performance of the *l*_1_-penalized *k*-class IV method. The RI property and ROC determine what subsets of cardinality *q* of columns of matrix A are in an orthonormal structure. These conditions are common in the high-dimensional setting of the linear model.

Definition 2.1: A matrix A has the RI property of order *q* if $(1-\Delta_{q})∥\delta {∥}_{2}^{2}\leq ∥A\delta {∥}_{2}^{2}\leq (1+\Delta_{q})∥\delta {∥}_{2}^{2}$ for all *q* -sparse vectors δ , where $\Delta_{q}\in (0,1)$ . To simplify the notation, we define

(2.3) $$\Delta_{q}^{-}(A)\|\delta {\|}_{2}^{2}\leq A\|\delta {\|}_{2}^{2}\leq \Delta_{q}^{+}(A)\|\delta {\|}_{2}^{2},\;\forall |\delta |\leq q,$$

where $\Delta_{q}^{+}(A)$ and $\Delta_{q}^{-}(A)$ are the upper and lower RI property constants of order *q*.

Definition 2.2. If $q+q^{\prime }\leq p$ , then $q,q^{\prime }\text{ - ROC}$ $\theta_{q,q^{\prime }}(A)$ is the smallest nonnegative number such that

$$|\langle A\delta ,A\delta^{\prime }\rangle |\leq \theta_{q,q^{\prime }}(A)\|\delta {\|}_{2}^{2}\|\delta^{\prime }{\|}_{2}^{2}$$

for all δ and $\delta^{\prime }$ , where δ and $\delta^{\prime }$ are *q*-sparse and *q*′-sparse vectors, respectively, and have nonoverlapping support.

## 3. *l*_1_-Penalized instrumental variables estimation

It is important to first state the conditions on which the *l*_1_-penalized IV estimation methods are based.

Assumption 1.

1.
$\left(Y_{i},X_{i},Z_{i.}:i=1,\ldots ,n\right)$ are independently and identically distributed;
2.
$E[Z_{i.}Z_{i.}^{T}]$ is of full rank and positive definite;
3.
$\left(\left(\begin{array}{c} e_{i}\\ \mu_{i} \end{array}\right)|Z_{i}\right)∼N(0,\Sigma )$ and $\Sigma =\left[\begin{array}{cc} \sigma_{e}^{2} & \sigma_{e\mu }\\ \sigma_{\mu e} & \sigma_{\mu }^{2} \end{array}\right]$ ;
4.
$\psi_{0}=(E[Z_{i.}Z_{i.}^{T}]{)}^{-1}E(Z_{i.}X_{i})$ with elements of $\psi_{0}$ being nonzero, i.e. $\psi_{0,j}\neq 0\forall j=1,\ldots ,L.$

Assumption 1.1 is a basic assumption that states that the observations are *i.i.d*. Assumption 1.2 requires the usual identification assumption to be satisfied and the matrix Z to be full rank. In assumption 1.3, we first make a conditional homoscedasticity assumption on the errors given the instruments, and we assume that the elements of Σ are finite.^27^ We relax assumption 1.3 and propose the robust methods in Section 3.4 by following Hausman et al.^20^ if the errors are heteroscedastic, which is more common in practical applications. Assumption 1.4 indicates that the matrix Z is associated with the exposure variable X **.**

The oracle class of IV estimators is found when the invalid instrumental variables $\left(Z_{IN}\right)$ are known, and we then set $Q_{IN}=\left[\begin{array}{cc} X & Z_{IN} \end{array}\right]$ . Specifically, we consider estimators of the form

(3.1) $${\hat{\Theta }}_{k}=\left(\begin{array}{c} \hat{\beta }\\ {\hat{\delta }}_{IN} \end{array}\right)=(Q_{IN}^{T}(I_{n}-kM_{\text{Z}})Q_{IN}{)}^{-1}Q_{IN}^{T}(I_{n}-kM_{\text{Z}})Y$$

with different methods of estimating *k*. Eq. (3.1) encompasses all of the well-known *k*-class estimators. For example, the OLS and TSLS estimators are special cases of these estimators when k=0 and k=1 , respectively. In addition, Eq. (3.1) corresponds to the LIML estimator when $k={\hat{\kappa }}_{\text{liml}}$ , where ${\hat{\kappa }}_{\text{liml}}$ is the smallest eigenvalue of the matrix $[W^{T}M_{\text{Z}}W{]}^{-1/2}W^{T}M_{Z_{IN}}W[W^{T}M_{Z}W{]}^{-1/2}$ , with $W=\left[\begin{array}{cc} Y & X \end{array}\right]$ , and therefore depends only on observable data and not on unknown parameters.^28^ The modification of the LIML method known as FUL^17^ is also classified as a *k*-class estimator where $k={\hat{\kappa }}_{\text{ful}}=[{\hat{\kappa }}_{\text{liml}}-C_{0}(1-{\hat{\kappa }}_{\text{liml}})/n]/[1-C_{0}(1-{\hat{\kappa }}_{\text{liml}})/n]$ with a constant value of $C_{0}$ . Note that ${\hat{\kappa }}_{\text{liml}}\geq 1$ since $\text{span}(Z_{IN})\subset \text{span}(Z)$ and $W^{T}M_{Z_{IN}}W$ cannot be smaller than $[W^{T}M_{Z}W{]}^{-1}$ when the number of invalid instruments is known. The FUL estimator was developed because the LIML estimator does not have moments since its distribution has heavy tails, leading to high dispersion in finite samples.^19^ The FUL estimator addresses this problem. This modification of LIML further leads to an FUL estimator with the existence of moments. LIML and FUL were developed as alternatives to the TSLS estimator since they are capable of handling weak instruments, many instruments and misspecification of the model.

### 3.1. Penalized k-class estimators

Here, we introduce the equivalent Lagrangian structure as an estimator of the causal effect, called the *penalized k-class IV (PKCIV)* estimation method, as follows:

(3.2) $$({\hat{\beta }}^{\left(\lambda \right)},{\hat{\delta }}^{\left(\lambda \right)})\in \underset{\beta ,\delta }{\text{argmin}}\;\frac{1}{2}(I_{n}-kM_{\text{Z}})(Y-X\beta -Z\delta ){\|}_{2}^{2}+\lambda \|\delta {\|}_{1}$$

for $\lambda \in R_{>0}$ . The class of estimators in (3.2) is a modification of the popular LASSO^29^ method, wherein we consider Model (2.1) and use $l_{1}$ -penalization to parameter δ with many valid and invalid instruments. The PKCIV method does not penalize $\beta_{0}$ because it is the main parameter of interest, and we do not wish to bias the estimation of the causal effect. The proposed estimator in (3.2) is a *k-class invalid and valid IV estimator* and can be seen as a generalization of Kang et al.'s^12^ estimator if k=1 , (3.2) is the penalized TSLS (PTSLS) estimator. Similarly, (3.2) corresponds to the penalized LIML (PLIML) and penalized FUL (PFUL) estimators when $k={\hat{\kappa }}_{\text{liml}}$ and $k={\hat{\kappa }}_{\text{ful}}$ , respectively.

**Figure 1.**
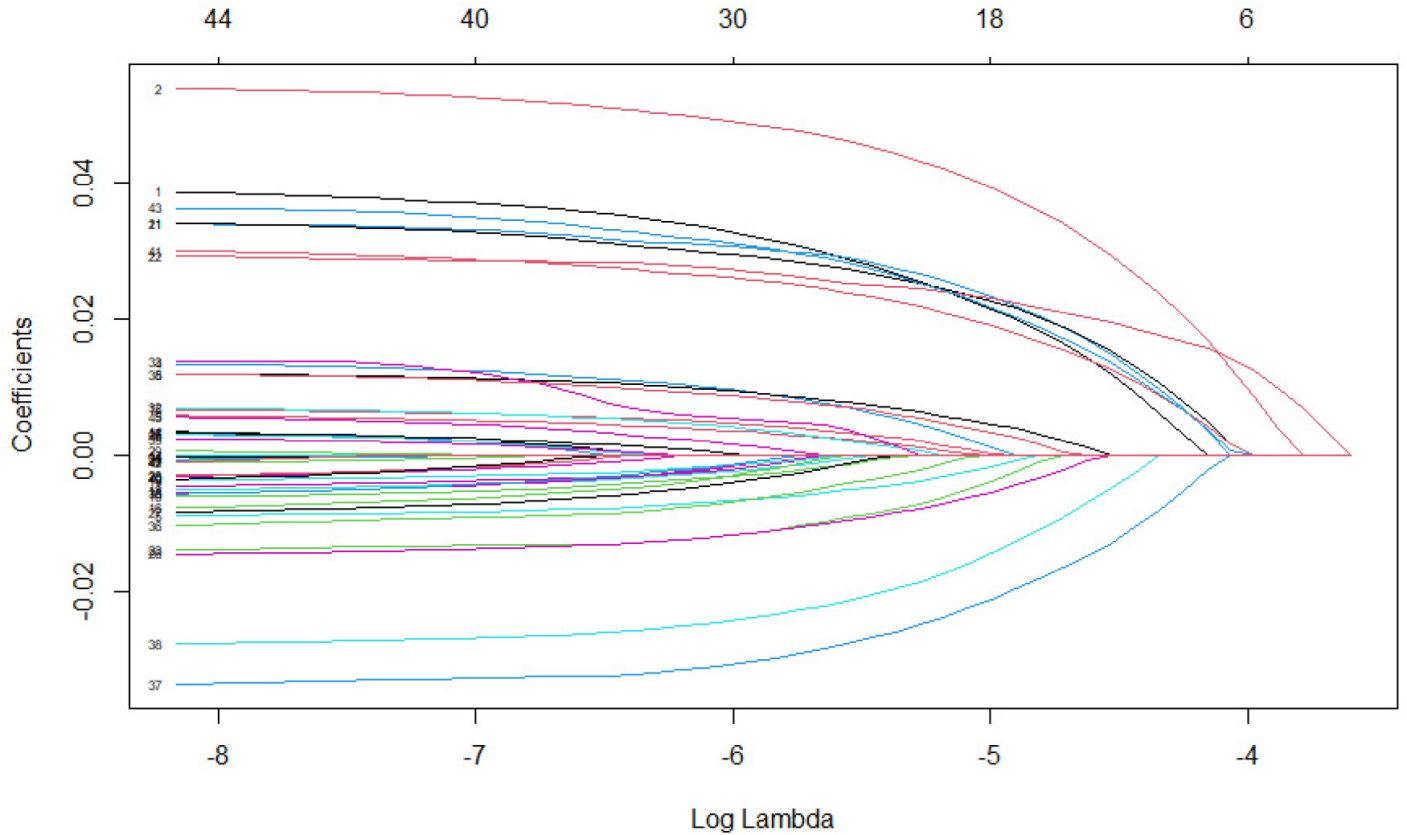
LASSO instrumental variable regularization path.

The choice of the tuning parameter λ affects the performance of the PKCIV estimator and affects the intensity of the sparsity of the solution. Figure 1 shows the LASSO regularization path using the IV method to illustrate how the coefficient estimates of δ decrease to zero as λ increases. Each curve corresponds to a variable. The axis above indicates the number of instruments at the current value of λ . For λ→0 , few elements of ${\hat{\delta }}^{\left(\lambda \right)}$ will be zero, indicating that most instruments are estimated to be invalid instruments. On the other hand, for large values of λ , the penalty function, $\lambda ∥\delta_{1}{∥}_{1}$ , surpasses the sum of squares, which strongly penalizes parameter δ , and most instruments are estimated as valid instruments. Intermediate tuning parameter values yield a balance between these two extremes. An important aspect of the PKCIV estimator is choosing the tuning parameter λ .

Several different methods for selecting λ have been discussed in the literature. Selecting λ through cross-validation is a very common data-driven approach that aims for optimal prediction accuracy. Various types of cross-validation exist, such as *K*-fold and leave-*out* cross-validation. In this paper, we use 10-fold cross-validation, which is frequently used in practice. We minimize the predictive error $\|Y-X\beta -Z\delta {\|}_{2}$ while using 10-fold cross-validation, and the parameter of interest is $\beta_{0}$ .

### 3.2. Estimating the causal effect

We introduce a numerical optimization algorithm for estimating parameters β and δ . The solution of the numerical algorithm is equivalent to the PKCIV estimator in (3.2). First, we rewrite (3.2) as

$${\hat{\beta }}^{\left(\lambda \right)},{\hat{\delta }}^{\left(\lambda \right)}=\underset{\beta ,\delta }{\text{argmin}}\;\frac{1}{2}\|(P_{\text{Z}}+(1-k)M_{\text{Z}})(Y-X\beta -Z\delta ){\|}_{2}^{2}+\lambda \|\delta {\|}_{1}.$$

Step-I: Then, we obtain the estimator ${\hat{\delta }}^{\left(\lambda \right)}$ for a given λ>0 as

$${\hat{\delta }}^{\left(\lambda \right)}=\underset{\delta }{\text{argmin}}\;\frac{1}{2}\|\tilde{Y}-\tilde{Z}\delta {\|}_{2}^{2}+\lambda \|\delta {\|}_{1},$$

where $\tilde{Y}=M_{\hat{X}}P_{\text{Z}}Y$ , $\tilde{Z}=M_{\hat{X}}Z$ and λ are estimated through cross-validation.

Step-II: Given the estimator ${\hat{\delta }}^{\left(\lambda \right)}$ , we obtain an estimator for β as

$${\hat{\beta }}^{\left(\lambda \right)}=\frac{{\tilde{X}}^{T}Y-{\hat{X}}^{T}Z{\hat{\delta }}^{\left(\lambda \right)}}{{\hat{X}}^{T}\hat{X}+d(X^{T}X-{\hat{X}}^{T}\hat{X})},$$

where $\tilde{X}=\hat{X}+d(X-\hat{X})$ and $d=(1-k{)}^{2}$ . Note that in the selection stage, we use the LASSO procedure with a *k*-class estimator-based objective function. The tuning parameter, λ, is chosen through cross-validation, wherein we minimize the predictive error for the PTSLS, PLIML and PFULL estimators. This algorithm uses 10-fold cross-validation to determine the optimal value of λ , selecting it on the basis of the cross-validation results. Each method in PKCIV provides both the estimated causal effect of exposure on the outcome and the set of invalid instruments for a specific λ . Finally, the algorithm gives a list of estimated results, which contains the estimations of δ , β , and the set of invalid instruments for the best λ . This numerical algorithm is thus simple and easy to calculate as least squares. The theoretical properties of this two-step algorithm are discussed in Appendix A. The PLIML estimator can be computed by finding ${\hat{\kappa }}_{\text{liml}}$ and then using this in the estimation of the causal effect of exposure on the outcome for $d=(1-{\hat{\kappa }}_{\text{liml}}{)}^{2}$ . Let $C_{0}=1$ ^17^ and ${\tilde{\kappa }}_{\text{ful}}=[{\hat{\kappa }}_{\text{liml}}-C_{0}(1-{\hat{\kappa }}_{\text{liml}})/n]/[1-C_{0}(1-{\hat{\kappa }}_{\text{liml}})/n]$ . Then, the value of ${\hat{\kappa }}_{\text{ful}}$ in step II is substituted for $d=(1-{\hat{\kappa }}_{\text{ful}}{)}^{2}$ to compute the PFUL estimator for the causal parameter.

### 3.3. Theoretical performance of the PKCIV estimator

To minimize the structure of the PKCIV method, Eq. (3.2) might have different minimizers, particularly for estimating the causal effect of parameter $\beta_{0}$ , because $\|\delta {\|}_{1}$ is not strictly convex. In this case, the value of the parameter may need to be carefully tuned to ensure that the algorithm is able to converge to the global minimum. The estimated difference between all the minimizers of (3.2) and $\beta_{0}$ , that is $\left|{\hat{\beta }}^{\left(\lambda \right)}-\beta_{0}\right|$ , is analyzed in this section. Through the RI property and ROC, we illustrate the performance of the PKCIV estimator in finite samples. Let $\hat{X}=P_{\text{Z}}X$ be the predicted value of X given Z and the residual-forming matrix be $M_{\text{Z}}$ . The solution of (3.2) is unique when the elements of the matrix $M_{\text{Z}}Z$ are taken from a continuous distribution.^30^ The following theorem is a generalization of the theorem based on PTSLS $\left({\hat{\beta }}^{\left(\lambda \right)}\right)$ provided by Kang et al.,^12^ wherein we consider the general estimator that includes the *k*-class IV methods.

Theorem 3.1. Consider model (2.1) with $\hat{X}=P_{\text{Z}}X$ under assumptions 1.1–1.4. Let ${\hat{\delta }}^{\left(\lambda \right)}$ and ${\hat{\beta }}^{\left(\lambda \right)}$ be the minimizers of (3.2) with $\left\{e\in R^{n}:Z^{T}M_{\hat{X}}P_{\text{Z}}e_{\infty }\leq \lambda /3\right\}$ for λ>0 . Then:

i. The estimator ${\hat{\beta }}^{\left(\lambda \right)}$ can be expressed as
(3.3) $${\hat{\beta }}^{\left(\lambda \right)}=\beta_{0}+\frac{{\hat{X}}^{T}P_{\text{Z}}P_{\hat{X}}Z(\delta_{0}-{\hat{\delta }}^{\left(\lambda \right)})+({\hat{X}}^{T}+d(X^{T}-{\hat{X}}^{T}))e}{\left\|\hat{X}{\|}_{2}^{2}+d(\|X{\|}_{2}^{2}-\|\hat{X}{\|}_{2}^{2}\right)};$$
ii. Suppose that the condition $\Delta_{2r}^{+}(Z)<2(\Delta_{2r}^{-}(Z)-\Delta_{2r}^{+}(P_{\hat{X}}Z))$ holds by definition of the RI constants. Then, ${\hat{\beta }}^{\left(\lambda \right)}$ is such that
(3.4) $$\|{\hat{\beta }}^{\left(\lambda \right)}-\beta_{0}{\|}_{2}\leq \frac{1}{{\left\|\hat{X}\right\|}_{2}+d({\left\|X\right\|}_{2}-{\left\|\hat{X}\right\|}_{2})}\left(\frac{4\lambda {\left(5\Delta_{2r}^{+}(P_{\hat{X}}Z)\right)}^{1/2}}{6(\Delta_{2r}^{-}(Z)-\Delta_{2r}^{+}(P_{\hat{X}}Z))-3\Delta_{2r}^{+}(Z)}\right)+\frac{\left|({\hat{X}}^{T}+d(X^{T}-{\hat{X}}^{T}))e\right|}{\left\|\hat{X}{\|}_{2}^{2}+d(\|X{\|}_{2}^{2}-\|\hat{X}{\|}_{2}^{2}\right)}$$

Proof
The first part of the theorem can be easily established by utilizing the algorithm primarily for estimating the causal effect. However, to guarantee the performance of the proposed method, the final part of the theorem must be proven. The proof of this theorem is presented in the Appendix.

Remark 1 The assumption $\Delta_{2r}^{+}(Z)<2(\Delta_{2r}^{-}(Z)-\Delta_{2r}^{+}(P_{\hat{X}}Z))$ in part (ii) of Theorem 3.1 involves the RI property constants, which are difficult to estimate. In addition to the RI property, the mutual incoherence property (MIP) is a commonly used condition in the sparse recovery literature. The MIP conditions are defined as

(3.5) $$\eta =\text{ma}{\text{x}}_{i\neq j}|\langle Z_{i}Z_{j}\rangle |,$$

which establishes the maximum pairwise correlation of the columns of the instrument's matrix Z , and the maximum strength of the individual instruments is measured as

(3.6) $$\rho =\text{ma}{\text{x}}_{j}\frac{\left|{\hat{X}}^{T}Z_{.j}\right|}{\left\|\hat{X}{\|}_{2}^{2}+d(\|X{\|}_{2}^{2}-\|\hat{X}{\|}_{2}^{2}\right)}.$$

The performance of the PKCIV is analyzed in terms of the MIP conditions in (3.5) and (3.6). We modify the bounds in (3.4) by following Corollary 2 in Kang et al.,^12^ wherein the number of invalid instruments is *r* such that r<min(1/12η,1/10ρ) . In addition, by rewriting the assumption $2(\Delta_{2r}^{-}(Z)-\Delta_{2r}^{+}(P_{\hat{X}}Z))-\Delta_{2r}^{+}(Z)>0$ in terms of two MIP constants η and ρ , under the conditions r<min(1/12η,1/10ρ) and η and ρ , the constraint from Lemma 3.1 can be modified and stated as

(3.7) $$\|{\hat{\beta }}^{\left(\lambda \right)}-\beta_{0}{\|}_{2}\leq \frac{1}{{\left\|\hat{X}\right\|}_{2}+d({\left\|X\right\|}_{2}-{\left\|\hat{X}\right\|}_{2})}\left(\frac{4\lambda \rho {\left(10(r+2r^{2}\eta )\right)}^{1/2}}{3-3r(6\eta +5\rho^{2})}\right)+\frac{\left|({\hat{X}}^{T}+d(X^{T}-{\hat{X}}^{T}))e\right|}{\left\|\hat{X}{\|}_{2}^{2}+d(\|X{\|}_{2}^{2}-\|\hat{X}{\|}_{2}^{2}\right)},$$

where $2(\Delta_{2r}^{-}(Z)-\Delta_{2r}^{+}(P_{\hat{X}}Z))-\Delta_{2r}^{+}(Z)\geq 1-r(6\eta +5\rho^{2})>0$ due to the upper and lower bounds of the RI property constants in terms of MIP conditions such as $\Delta_{2r}^{-}(Z)\leq 1+\mu (2r-1)$ , $\Delta_{2r}^{+}(Z)\geq 1-\eta (2r-1)$ , $\Delta_{2r}^{+}(P_{\hat{X}}Z)\leq 2r\rho^{2}\Delta_{2r}^{+}(Z)$ , and $\Delta_{2r}^{-}(P_{\hat{X}}Z)\leq 2r\rho^{2}\Delta_{2r}^{-}(Z)$ .

### 3.4. LASSO-type jackknife instrumental variable estimation

The LASSO procedure for IV estimation for some valid and invalid instruments was proposed by Kang et al.^12^ It is known as the PTSLS estimator, which is a special form of the PKCIV estimator when k=1 . The PTSLS estimators of δ and β can be computed in two parts. The PTSLS estimator of δ **,** for a given λ>0 , from (3.2) is defined as

(3.8) $${\hat{\delta }}^{\left(\lambda \right)}=\underset{\delta }{\text{argmin}}\;\frac{1}{2}\|\tilde{Y}-\tilde{Z}\delta {\|}_{2}^{2}+\lambda \|\delta {\|}_{1}.$$

The matrix $\tilde{Z}$ in (3.8) depends on $\hat{X}$ , which is estimated from the first-stage regression; thus, the bias of TSLS depends on $E[{\hat{X}}^{T}e]$ . For observation *i*,

$$E[{\hat{X}}^{T}e]=E[{\hat{\psi }}^{T}Z_{i.}^{T}e_{i}]=E[E\{({\hat{\psi }}^{T}Z_{i.}^{T}e_{i}+\mu_{i}^{T}Z_{i.}{\left(Z^{T}Z\right)}^{-1}Z_{i.}^{T}e_{i})|Z\}]=\frac{L}{n}\sigma_{e\mu },$$

where $\sigma_{e\mu }$ measures the degree of endogeneity. $\frac{L}{n}\sigma_{e\mu }$ arises from the correlation of ${\hat{X}}_{i}$ for observation *i* with $e_{i}$ . In addition, this bias continues even if all the valid instruments are uncorrelated with $e_{i}$ . This becomes a more serious problem in the presence of many or weak instruments, which increases the bias of the PTSLS estimator.^7^ Another issue with the TSLS, as shown by Hausman et al.^20^ and Bekker,^31^ is that with many (weak) instruments, the TSLS is not consistent, even under homoscedasticity. The LIML and FUL estimators are efficient with many weak instruments and under homoscedasticity. However, these *k*-class IV methods are not robust when the data are heteroscedastic. This prompts us to introduce a new class of LASSO-type jackknife IV estimator (LJIVE) that is robust to heteroscedasticity and many instruments by following Hausman et al.^20^ The leave-one-out procedure in IVs regression can reduce bias by systematically excluding each observation, performing the estimation, and then aggregating the results. The penalized jackknife TSLS (PJTSLS), penalized jackknife LIML (PJLIML), and penalized jackknife FUL (PJFUL) are all members of a class of LJIVE.

Lemma 3.3 ^7^ Let $X_{\left(-i\right)}$ be an (n−1)×1 vector given by X with the *i*th row removed and, similarly, $Z_{\left(-i\right)}$ be an (n−1)×L matrix. The *i*th row removes the dependence of the composing instrument on the exposure variable so that

$$E[{\hat{\psi }}_{\left(-i\right)}^{T}Z_{i.}^{T}e_{i}]=0.$$

**Figure 2.**
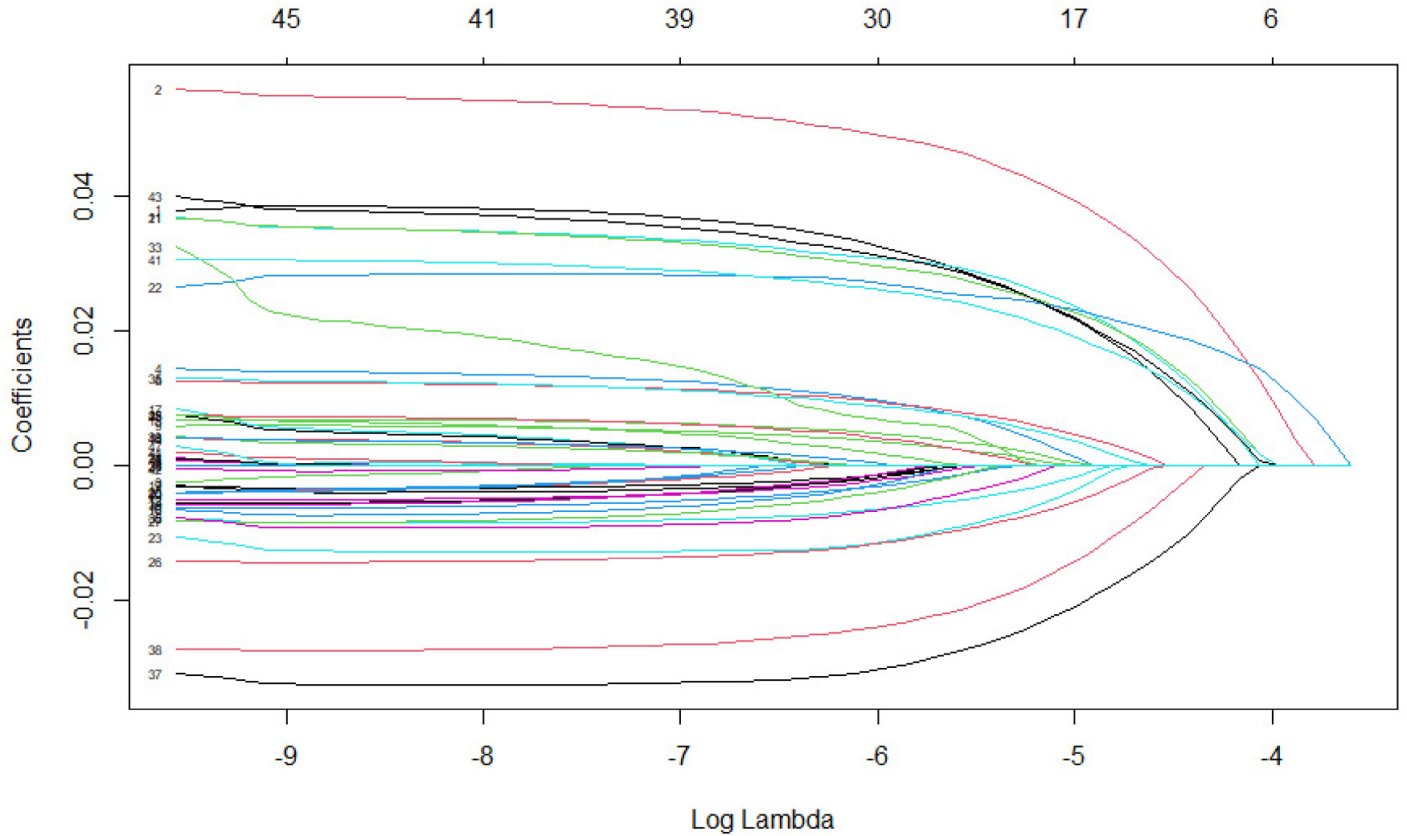
LASSO jackknife instrumental variable regularization path.

Proof of Lemma 3.3 is provided in the appendix. We estimate the fitted value of exposure via Lemma 3.3 such that ${\hat{X}}_{\text{jiv}}$ is the n×1 vector with the *i*th * *row of $Z_{i.}{\hat{\psi }}_{\left(-i\right)}$ , where ${\hat{\psi }}_{\left(-i\right)}$ is well defined in the proof of Lemma 3.3 in Appendix C. Formally, the LJIVE for δ is obtained for a given λ>0 as

(3.9) $${\hat{\delta }}_{\text{jiv}}^{\left(\lambda \right)}=\underset{\delta }{\text{argmin}}\;\frac{1}{2}\|\tilde{Y}-{\tilde{Z}}_{\text{jiv}}\delta {\|}_{2}^{2}+\lambda \|\delta {\|}_{1},$$

where $\tilde{Y}=M_{{\hat{X}}_{\text{jiv}}}P_{\text{Z}}Y$ , ${\tilde{Z}}_{\text{jiv}}=M_{{\hat{X}}_{\text{jiv}}}Z$ . The LJIVE for β using ${\hat{\delta }}_{\text{jiv}}^{\left(\lambda \right)}$ in (3.9) is defined as

(3.10) $${\hat{\beta }}_{\text{jiv}}^{\left(\lambda \right)}=\frac{{\tilde{X}}_{\text{jiv}}^{T}Y-{\hat{X}}_{\text{jiv}}^{T}{Z\hat{\delta }}_{\text{jiv}}^{\left(\lambda \right)}}{{\hat{X}}_{\text{jiv}}^{T}{\hat{X}}_{\text{jiv}}+d(X^{T}X-{\hat{X}}_{\text{jiv}}^{T}{\hat{X}}_{\text{jiv}})},$$

where ${\tilde{X}}_{\text{jiv}}^{T}={\hat{X}}_{\text{jiv}}+d_{\text{jiv}}(X-{\hat{X}}_{\text{jiv}})$ and $d_{\text{jiv}}=(1-k_{\text{jiv}}{)}^{2}$ . PJTSLS $\left({\hat{\beta }}_{\text{jiv}}^{\left(\lambda \right)}\right)$ occurs with $k_{\text{jiv}}=1$ , PJLIML $\left({\hat{\beta }}_{\text{jiv}}^{\left(\lambda \right)}\right)$ uses $k_{\text{jiv}}={\overset{ˇ}{\kappa }}_{\text{liml}}$ , and PJFUL $\left({\hat{\beta }}_{\text{jiv}}^{\left(\lambda \right)}\right)$ arises with $k_{\text{jiv}}={\overset{ˇ}{\kappa }}_{\text{ful}}$ . ${\hat{\beta }}_{\text{jiv}}^{\left(\lambda \right)}$ can also be viewed as another estimator by setting $k_{\text{jiv}}=0$ . For PJLIML, $k_{\text{jiv}}={\overset{ˇ}{\kappa }}_{\text{liml}}$ is estimated, where ${\overset{ˇ}{\kappa }}_{\text{liml}}$ is the smallest eigenvalue^20^ of the matrix $(W^{T}W{)}^{-1}\left(W^{T}P_{\text{Z}}W-\overset{n}{\underset{i=1}{\sum }}\;P_{ii}W_{i}W_{i}^{T}\right)$ , with $W=\left[\begin{array}{cc} Y & X \end{array}\right]$ , and, for PJFUL, $k_{\text{jiv}}={\overset{ˇ}{\kappa }}_{\text{ful}}=\left[{\overset{ˇ}{\kappa }}_{\text{liml}}-\left(1-{\overset{ˇ}{\kappa }}_{\text{liml}}\right)/n\right]/\left[1-\left(1-{\overset{ˇ}{\kappa }}_{\text{liml}}\right)/n\right]$ . The tuning parameter, λ, is chosen through 10-fold cross-validation, wherein we minimize the predictive error for the PJTSLS, PJLIML and PJFULL estimators. We display the solution path of the LASSO-based jackknife IV method in Figure 2 to visualize the impact of the penalty parameter λ on the estimated ${\hat{\delta }}_{\text{jiv}}^{\left(\lambda \right)}$ . Tibshirani^29^ proposed the LASSO estimator for classical linear regression. The LASSO estimates are nonlinear and nondifferentiable functions of the outcome values, making accurate estimation of their standard errors difficult. As an alternative, Tibshirani^29^ suggested the use of bootstrapping to calculate the standard error. Bootstrap methods are commonly used in statistics and econometrics, as well as in Mendelian randomization (see, e.g. Refs.^32,33^). Therefore, the standard error and confidence intervals of the proposed methods and PTSLS can be estimated by bootstrapping.

Remark 2 The theoretical performance of the LJIVE can be generalized on the basis of Theorem 3.1 via the estimator ${\hat{\beta }}_{\text{jiv}}^{\left(\lambda \right)}$ . When we remove the dependence of the constructed instruments on the exposure variable for observation *i*, we use ${\hat{\psi }}_{\left(-i\right)}=(Z_{\left(-i\right)}^{T}Z_{\left(-i\right)}{)}^{-1}Z_{\left(-i\right)}^{T}X_{\left(-i\right)}$ instead of $\hat{\psi }=(Z^{T}Z{)}^{-1}Z^{T}X$ *.* This implies that ${\tilde{X}}_{\text{jiv}}^{T}={\hat{X}}_{\text{jiv}}+d(X-{\hat{X}}_{\text{jiv}})$ *.* We then replace $\tilde{X}$ with ${\tilde{X}}_{\text{jiv}}$ in (3.7) to obtain the estimation error bounds for the LJIVE, ${\hat{\beta }}_{\text{jiv}}^{\left(\lambda \right)}$ , as

$$\|{\hat{\beta }}_{\text{jiv}}^{\left(\lambda \right)}-\beta_{0}{\|}_{2}\leq \frac{\left|({\tilde{X}}_{\text{jiv}}^{T}+d(X^{T}-{\tilde{X}}_{\text{jiv}}^{T}))e\right|}{\left\|{\hat{X}}_{\text{jiv}}{\|}_{2}^{2}+d(\|X{\|}_{2}^{2}-\|{\hat{X}}_{\text{jiv}}{\|}_{2}^{2}\right)}+\frac{\left((4\lambda \rho_{\text{jiv}}{\left(10(r+2r^{2}\eta )\right)}^{1/2})/(3-3r(6\eta +5\rho_{\text{jiv}}^{2}))\right)}{\left\|{\hat{X}}_{\text{jiv}}{\|}_{2}^{2}+d(\|X{\|}_{2}^{2}-\|{\hat{X}}_{\text{jiv}}{\|}_{2}^{2}\right)},$$

under $\Delta_{2r}^{+}(Z)<2(\Delta_{2r}^{-}(Z)-\Delta_{2r}^{+}(P_{{\hat{X}}_{\text{jiv}}}Z))$ , where $\rho_{\text{jiv}}=\text{ma}{\text{x}}_{j}\frac{\left|{\tilde{X}}_{\text{jiv}}^{T}Z_{.j}\right|}{\left\|{\hat{X}}_{\text{jiv}}{\|}_{2}^{2}+d(\|X{\|}_{2}^{2}-\|{\hat{X}}_{\text{jiv}}{\|}_{2}^{2}\right)}$ .

## 4. Empirical study

We consider two experimental designs to examine the finite-sample behavior of the proposed estimators through Monte Carlo simulations. The objective of Model-I design is to assess the performance of the PLIML and PFUL estimators in the presence of numerous weak instruments and, subsequently, their performances with those of PTSLS. The objective of Model-II design is to evaluate the performance of all estimators in the presence of heteroscedastic errors.

***Model I:*** We begin with a model in which the first-stage regression model is linear, and the errors are homoscedastic in the form:

(4.1) $$\begin{array}{rl} Y_{i} & =X_{i}\beta_{0}+Z_{i.}^{T}\delta_{0}+e_{i},\\ X_{i} & =Z_{i.}^{T}\psi_{0}+\mu_{i}, \end{array}$$

where

$$\left(\begin{array}{c} e_{i}\\ \mu_{i} \end{array}\right)\overset{i.i.d}{∼}\;N\left(\left[\begin{array}{c} 0\\ 0 \end{array}\right],\left[\begin{array}{cc} \sigma_{e}^{2} & \sigma_{e\mu }\\ \sigma_{\mu e} & \sigma_{\mu }^{2} \end{array}\right]\right)$$

with $\sigma_{e}^{2}=\sigma_{\mu }^{2}=1$ , and instrumental variables $Z_{i.}$ are drawn from the multivariate normal distribution, i.e. $Z_{i.}\overset{i.i.d}{∼}\;N(0,\Sigma_{\text{z}})$ , with $\Sigma_{\text{z}}=\text{diag}(\sigma_{1}^{2},\ldots ,\sigma_{L}^{2})$ by setting all the diagonal elements as one and the off-diagonal elements as η , which is a pairwise correlation between instruments. Three different values of η, η=0.30,0.60 and 0.75 are set to consider weak, moderate and strong correlations between instruments. We set parameters $\beta_{0}=1$ , ${\psi_{0}}_{j}=0.10$ , and $\delta_{0}=(1_{0.3L},0_{0.7L}{)}^{T}$ , where we change *r* by increasing the number of instruments (L) in $∥\delta_{0}{∥}_{0}=r$ , and the causal parameter $\beta_{0}$ is the quantity of interest. The degree of endogeneity is measured by $\sigma_{e\mu }$ , wherein we set the values of $\sigma_{e\mu }$ from 0.30 to 0.90, while $\sigma_{e\mu }=0$ represents no endogeneity. We set the sample sizes to 200 , 500 and 1000. We consider cases with different numbers of instruments to assess the performance of the proposed estimators with many weak and invalid instruments. The total number of instruments (L) is selected by varying 10% to 70% of the sample size in a 10% interval; for example, *L* ranges from 20 to 140 when the sample size n=200 . Increasing *L* from 50% to 70% corresponds to the high-dimensional setting case.

***Model II:*** The data generation process of the second model is given by $Y_{i}=X_{i}\beta_{0}+Z_{i.}^{T}\delta_{0}+e_{i}$ and $X_{i}=Z_{i.}^{T}\psi_{0}+\mu_{i}$ , where the true parameter $\left(\beta_{0},\delta_{0}\right)$ values remain the same as those in Model (4.1) and $Z_{i.}\overset{i.i.d}{∼}\;N(0,I_{L})$ , where L∈{15,30,60} and *r* represent the invalid instruments by setting 30% of *L* rounded to the nearest whole number. We set $\vartheta^{2}=\sigma_{\mu }^{-2}\{{\left(Z\psi_{0}\right)}^{T}Z\psi_{0}\}$ , where $\vartheta^{2}$ is intimately related to the concentration parameter (CP). We consider $\vartheta^{2}=8$ and $\vartheta^{2}=64$ to vary the strength of the instruments.^34^ Both values of CP represent weak instruments and the lower the value of the CP parameter the weaker the instruments are. The value of ${\psi_{0}}_{j}$ is selected on the basis of the parameter $\vartheta^{2}$ .^2^ The CP measures the strength of the instruments, and it is also the first-stage *F* statistic when all the instruments are valid.^35^ The parameter $\vartheta^{2}$ increases at the same level as the sample size (n) , i.e. $\vartheta^{2}$ approaches $n\vartheta_{0}^{2}$ for some $\vartheta_{0}^{2}>0$ . We set *n* to 200, 500, 1000 and 5000. For Model-II we included 5000 observations to reflect the larger sample sizes usually available in modern MR analysis. Due to the high computational cost, we used only sample sizes of 200 to 1000 for Model-I. The second model is similar to the first model, but the errors are not homoscedastic. The errors are allowed to be heteroscedastic by following the design of Matsushita and Otsu.^36^ However, the disturbance terms $e_{i}$ and $\mu_{i}$ are generated as $(e_{i},\mu_{i})=\left\{\left(1+\phi \overset{L}{\underset{j=r+1}{\sum }}\;Z_{ij}\right)\varepsilon_{1i},\sigma_{e\mu }\mu_{i}+{\left(1-\sigma_{e\mu }^{2}\right)}^{1/2}\varepsilon_{2i}\right\}$ , where $\varepsilon_{1i}$ and $\varepsilon_{2i}$ are drawn from the normal distribution and where $\varepsilon_{1i},\varepsilon_{2i}\overset{i.i.d}{∼}\;N(0,1)$ , $\sigma_{e\mu }\in \{0.3,0.6\}$ and ϕ∈{0,0.30} are drawn for the homoscedastic and heteroscedastic error cases, respectively^36^ and.^37^ We consider the errors to be heteroscedastic and homoscedastic to gain a broader view of the performances of the estimators. A total of 1000 Monte Carlo replications are used for each experiment.

### 4.1. Simulation results

**Figure 3.**
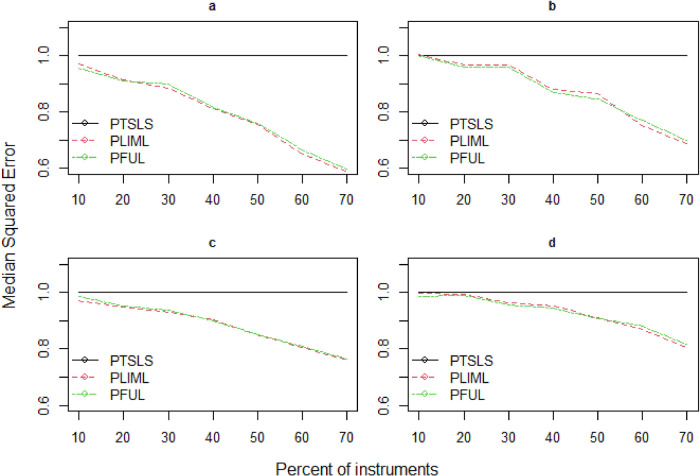
Relative median squared errors of PTSLS, PLIML and PFUL vs. percent of instruments×n when the sample size is 200 and (a) low endogeneity and low correlation exist between instruments, (b) low endogeneity and high correlation exist between instruments, (c) high endogeneity and low correlation exist between instruments, and (d) high endogeneity and high correlation exist between instruments.

**Figure 4.**
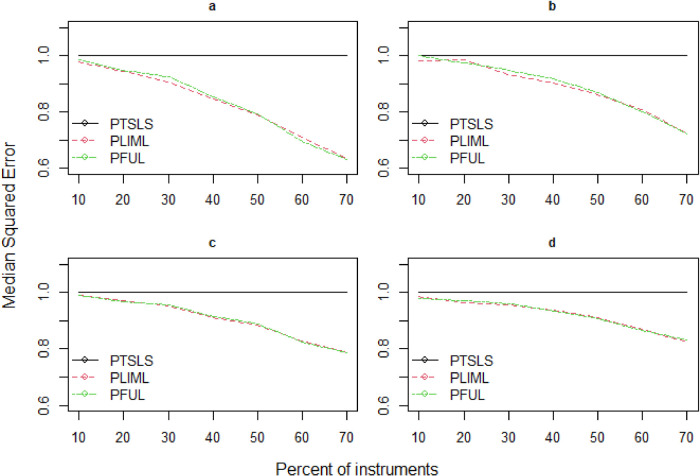
Relative median squared errors of PTSLS, PLIML and PFUL vs. percent of instruments×n when the sample size is 500 and (a) low endogeneity and low correlation exist between instruments, (b) low endogeneity and high correlation exist between instruments, (c) high endogeneity and low correlation exist between instruments, and (d) high endogeneity and high correlation exist between instruments.

**Figure 5.**
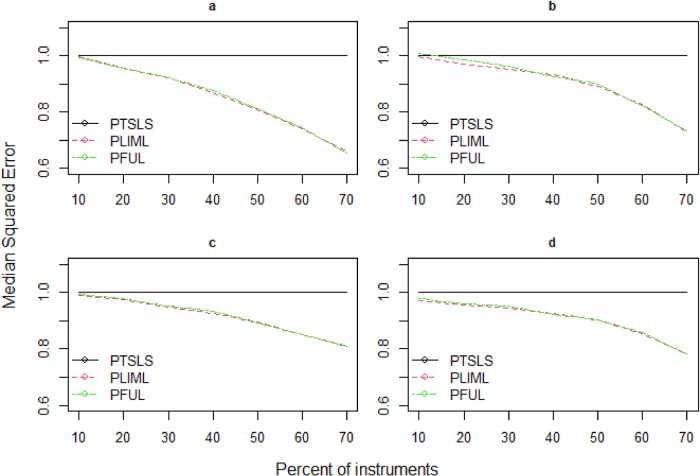
Relative median squared errors of PTSLS, PLIML and PFUL vs. percent of instruments×n when the sample size is 1000 and (a) low endogeneity and low correlation exist between instruments, (b) low endogeneity and high correlation exist between instruments, (c) high endogeneity and low correlation exist between instruments, and (d) high endogeneity and high correlation exist between instruments.

***Model I:*** We examine the PTSLS, PLIML and PFUL estimators for the first model in (4.1). We replicate the simulation study of Kang et al.^12^ and propose robust estimators (PLIML and PFUL) to overcome the large bias relative to standard errors when many weak valid and invalid instruments are present. The mean squared error is not a standard comparison in this situation because LIML endures the moment problem, and high dispersion relates to the lack of moments in LIML; as a result, we instead report the median squared error (MSE). Figures 3–5 depict the estimated results of the PKCIV estimators (PTSLS, PLIML and PFUL) of $\beta_{0}$ in terms of the relative median squared error^2^ and number of instruments for sample sizes of n=200 , n=500 and n=1000 . In each figure, we fix the sample size and increase the number of instruments to observe the performances of the proposed estimators (PLIML and PFUL) and the PTSLS^12^ estimator with many weak and invalid IVs. In addition, the numbers of invalid instruments (r) and valid instruments (L−r) increase with the total number of instruments. This is true from low- to high-dimensional settings, where L=0.1n to L=0.7n , respectively. The PLIML and PFUL estimators perform better as the number of valid and invalid weak instruments increases. The performances of the PLIML and FUL estimators are almost equivalent for many instruments; these results align with those of Hahn et al.^19^ However, neither FUL nor LIML dominate each other in practice. Figures 3–5 (b) show that the median squared errors of the PLIML and PFUL estimators are slightly greater than those of the PTSLS estimator when the number of instruments is 10% of the sample size. Table 1 indicates the results of the rate of decrease (%) to examine the relative decrease in median squared error due to sample size. As the sample size increases, the rate of decrease increases, and the performance of the proposed estimators improves. Overall, these simulation results demonstrate that the proposed PLIML and PFUL estimators perform better than PTSLS in the case of many instruments in terms of median squared errors.

**Table 1.** Rate of decrease (%) for sample size using the relative median squared error.

*L* (%)	PTSLS	PLIML	PFUL	PTSLS	PLIML	PFUL	PTSLS	PLIML	PFUL	PTSLS	PLIML	PFUL
	$\sigma_{e\mu }=0.30$ and η=0.30			$\sigma_{e\mu }=0.30$ and η=0.60			$\sigma_{e\mu }=0.60$ and η=0.30			$\sigma_{e\mu }=0.60$ and η=0.60		
*Sample size 200 to 500*												
10	9.00	8.23	6.34	12.31	14.1	12.50	−3.54	−5.68	−4.01	5.95	5.67	5.04
20	12.85	9.97	9.29	17.76	16.28	16.39	2.17	0.18	0.73	7.41	8.98	7.71
30	16.14	14.03	13.92	13.49	16.47	14.50	6.02	3.52	4.24	6.99	5.98	5.39
40	17.96	14.33	14.11	18.83	16.67	14.38	6.47	5.50	4.94	5.94	6.18	5.62
50	16.66	13.04	13.07	13.83	14.1	11.57	8.61	4.66	4.52	6.91	5.33	5.01
60	17.32	9.54	13.68	17.83	11.86	14.67	6.80	4.11	5.00	7.91	6.24	7.87
70	15.73	9.30	11.22	16.25	11.57	13.83	6.94	3.74	4.11	8.46	4.68	4.97
*Sample size 200 to 1000*												
10	20.15	17.99	17.24	24.78	25.40	24.32	1.17	−0.70	0.27	11.32	10.82	9.00
20	24.28	21.08	20.50	28.48	28.26	26.63	8.72	6.23	6.22	14.28	14.91	14.09
30	27.52	24.38	25.89	25.22	26.30	24.81	11.82	9.82	10.56	13.09	11.59	10.68
40	26.46	21.45	21.17	28.69	24.56	24.07	11.85	9.54	8.57	12.31	11.84	11.48
50	27.87	22.98	23.09	26.17	23.74	21.67	14.25	9.66	10.06	13.86	11.48	10.95
60	28.49	18.27	20.12	27.76	20.48	22.68	13.39	8.46	8.87	14.15	12.32	13.06
70	26.06	16.99	19.03	25.32	20.88	21.93	12.46	7.21	7.53	14.04	12.97	14.01

Note: PTSLS = “Penalized two-stage least square”^12^; proposed estimators: PLIML = “Penalized limited information maximum likelihood”; PFUL = “Penalized FUL.”^17^

***Model II:*** Tables 2a, 2b, 2c, 3a, 3b, and 3c present the simulation results in terms of median bias, MSE and average standard errors for oracle-LIML (OLIML),^3^ naive-LIML (NLIML),^4^ oracle-FUL (OFUL), naive-LIML (NFUL), penalized *k*-class IV estimators (PTSLS, PLIML, PFUL) and LASSO-type jackknife IV estimators (PJTSLS, PJLIML, PJFUL) for a range of numbers of instruments *L*, the degree of endogeneity $\sigma_{e\mu }$ , the sample size *n*, and the strength of the instruments $\vartheta^{2}$ . The standard errors for the penalized methods are calculated by bootstrapping with 500 resamples. The average standard error performance criterion has been widely used in previous MR simulation studies, such as those by Burgess et al.^38^ Tables 2a, 2b, 2c, 3a, 3b, and 3c present the results when the errors are heteroscedastic and homoscedastic, respectively. We estimate the causal effect for each experiment and the penalization parameter λ in the LASSO procedures selected by 10-fold cross-validation. The results of the OLIML and OFUL estimators are based on knowing which instruments are invalid with $\text{supp}(\delta_{0})$ , and the results of the NLIML and NFUL estimators are based on not knowing which instruments are invalid. We expect NLIML and NFUL to perform poorly in the presence of invalid instruments.^39^ The PTSLS estimator is taken from the *sisVIVE* routine in the literature.^12^ As discussed earlier, the PLIML and PFUL estimators are robust and viable alternatives to PTSLS (*sisVIVE*) when there are many weak instruments. However, PLIML and PFUL can be inconsistent in terms of many instruments and heteroskedasticity. Therefore, we present the results of PJTSLS, PJLIML and PJFUL proposed for reducing the bias caused by the endogeneity, weak instruments and heteroscedastic errors in the IV model with invalid instruments.

The results in Table 2a when L=15 and r=5 show some interesting patterns. The PJTSLS estimator outperforms the other LASSO procedures (PTSLS, PLIML, PFUL, PJLIML and PJFUL) in terms of bias and MSE. However, the PJLIML and PJFUL estimators are more efficient, with estimates having lower mean standard errors than those of the other methods. The performance of the estimators improves when the sample size is increased, excluding the NLIML and NFUL estimators, because of the number of invalid instruments. In the presence of heteroscedasticity, the MSE of the estimators is greater than that in the homoscedastic scenario. The bias, MSE and mean standard error values of the estimators decrease when the parameter $\vartheta^{2}$ is changed from 8 to 64. $\vartheta^{2}=2$ represents the case in which the instruments are very weak, and the proposed estimators are more robust in this situation. Note that the OLIML and OFUL methods do not perform well in the presence of weak instruments and heteroscedasticity. This might be because the LIML and FUL methods are not consistent in handling this situation.^20^ The PJLIML and PJFUL methods exhibit greater bias and MSE than PTSLS when $\sigma_{e\mu }=0.60$ and $\vartheta^{2}=64$ . This is the case when the instruments are slightly strong; however, in this situation, the alternative choice is PJTSLS, which is efficient. When *L* increases from 15 to 30 (Table 2b), PJLIM and PJFUL outperform in a certain case, such as when n=200 , $\sigma_{e\mu }=0.60$ and $\vartheta^{2}=64$ . Table 2b and 2c present the estimation results for L=30 and L=60 , respectively. The bias, MSE and mean standard error increase for all IV methods when the number of instruments is 30 or greater. However, in these situations, the use of LASSO-type jackknife IV estimators improves the estimation of the causal effect in the MR. In addition, we observe that the PJTSLS outperforms all other estimators where the LASSO procedure is used for the estimation of IVs when the errors are heteroscedastic.

**Table 2a.** Estimation results of the estimators for *L* = 15 and *r* = 5 with heteroscedastic errors.

	*n* = 200			*n* = 500			*n* = 1000			*n* = 5000		
Estimators	Bias	MSE	SE	Bias	MSE	SE	Bias	MSE	SE	Bias	MSE	SE
$\sigma_{\mu e}=0.30$												
$\vartheta^{2}=8$												
OLIML	0.7340	0.5388	28.908	0.7405	0.5483	20.033	0.6888	0.4745	5.1437	0.8116	0.6587	58.142
NLIML	20.611	424.81	420.72	31.456	989.46	962.48	44.306	1963.0	1511.7	101.65	10332	4629.0
OFUL	0.5688	0.3236	0.6942	0.5985	0.3582	0.7159	0.5590	0.3125	0.7127	0.6198	0.3841	0.9412
NFUL	12.756	162.73	7.2819	20.095	403.79	10.919	28.273	799.35	15.620	65.525	4293.5	29.454
PTSLS	0.8313	0.6910	0.6360	0.7855	0.6170	0.8328	0.7478	0.5592	1.0125	0.7797	0.6079	3.6787
PLIML	0.4367	0.1907	0.1634	0.4012	0.1609	0.0913	0.3754	0.1410	0.0604	0.3687	0.1359	0.0251
PFUL	0.4366	0.1907	0.1625	0.4012	0.1610	0.0913	0.3743	0.1401	0.0603	0.3687	0.1360	0.0255
PJTSLS	0.3967	0.1573	0.4436	0.3868	0.1496	0.4483	0.3742	0.1400	0.4556	0.3240	0.1050	0.6356
PJLIML	0.4056	0.1646	0.1195	0.3925	0.1540	0.0709	0.3704	0.1372	0.0478	0.3682	0.1356	0.0215
PJFUL	0.4059	0.1648	0.1191	0.3911	0.1529	0.0708	0.3709	0.1375	0.0477	0.3681	0.1355	0.0215
$\vartheta^{2}=64$												
OLIML	0.2136	0.0456	0.2315	0.2124	0.0451	0.2297	0.2115	0.0447	0.2351	0.2155	0.0465	0.3251
NLIML	7.1232	50.740	2.6997	11.334	128.45	3.5642	15.984	255.50	4.2924	34.666	1201.7	10.402
OFUL	0.2106	0.0443	0.2268	0.2079	0.0432	0.2250	0.2085	0.0435	0.2300	0.2097	0.0440	0.3119
NFUL	6.7773	45.932	2.1058	10.783	116.28	3.0155	15.162	229.90	3.7798	33.002	1089.1	7.8571
PTSLS	0.5964	0.3557	0.3906	0.5810	0.3376	0.4541	0.5693	0.3241	0.6262	0.5242	0.2748	1.1634
PLIML	0.4669	0.2180	0.1930	0.4176	0.1744	0.1014	0.3850	0.1482	0.0734	0.3704	0.1372	0.0264
PFUL	0.4683	0.2193	0.1916	0.4171	0.1739	0.1012	0.3867	0.1496	0.0733	0.3706	0.1374	0.0263
PJTSLS	0.3682	0.1355	0.2318	0.3652	0.1333	0.2396	0.3590	0.1289	0.2476	0.3026	0.0916	0.2298
PJLIML	0.4126	0.1702	0.1312	0.4036	0.1629	0.0738	0.3808	0.1450	0.0496	0.3699	0.1369	0.0221
PJFUL	0.4178	0.1746	0.1309	0.4040	0.1632	0.0736	0.3801	0.1445	0.0495	0.3698	0.1368	0.0221
$\sigma_{\mu e}=0.60$												
$\vartheta^{2}=8$												
OLIML	0.6836	0.4673	15.864	0.6761	0.4572	17.651	0.6360	0.4045	10.821	0.6391	0.4085	12.112
NLIML	20.282	411.36	693.37	31.322	981.08	428.43	45.221	2045.0	921.29	91.826	8432.1	1456.8
OFUL	0.4786	0.2291	0.5509	0.4757	0.2263	0.5939	0.4871	0.2372	0.5424	0.4926	0.2426	0.6636
NFUL	12.025	144.61	6.7822	18.742	351.27	10.349	26.360	694.84	14.310	57.545	3311.5	29.625
PTSLS	0.9737	0.9481	0.4810	0.9451	0.8932	0.6228	0.9519	0.9061	0.7168	0.9769	0.9544	2.8269
PLIML	0.8076	0.6523	0.1221	0.7891	0.6226	0.0703	0.8636	0.7457	0.0455	0.8620	0.7430	0.0204
PFUL	0.8071	0.6514	0.1215	0.7887	0.6221	0.0702	0.8626	0.7441	0.0454	0.8618	0.7428	0.0203
PJTSLS	0.5242	0.2748	0.4399	0.5105	0.2606	0.4547	0.5132	0.2634	0.4467	0.4641	0.2154	0.7887
PJLIML	0.7806	0.6093	0.0888	0.7801	0.6085	0.0549	0.8612	0.7416	0.0379	0.8614	0.7420	0.0168
PJFUL	0.7825	0.6123	0.0886	0.7795	0.6077	0.0548	0.8611	0.7416	0.0379	0.8613	0.7418	0.0169
$\vartheta^{2}=64$												
OLIML	0.2073	0.0430	0.2538	0.2020	0.0408	0.2464	0.1843	0.0340	0.2773	0.2086	0.0435	0.4208
NLIML	8.7991	77.423	50.054	12.303	151.37	5.4473	16.880	284.92	7.5591	36.068	1300.9	240.52
OFUL	0.2004	0.0401	0.2374	0.1983	0.0393	0.2296	0.1824	0.0333	0.2541	0.2038	0.0415	0.3585
NFUL	7.9965	63.944	3.4328	11.525	132.82	3.9451	15.7973	249.55	5.0733	33.817	1143.6	8.7772
PTSLS	0.6690	0.4476	0.3561	0.6541	0.4279	0.4717	0.6672	0.4452	0.7024	0.6319	0.3993	2.1266
PLIML	0.7147	0.5108	0.1601	0.7503	0.5629	0.0896	0.8447	0.7136	0.0634	0.8584	0.7369	0.0252
PFUL	0.7144	0.5103	0.1597	0.7520	0.5655	0.0894	0.8458	0.7154	0.0633	0.8585	0.7370	0.0254
PJTSLS	0.4722	0.2229	0.2134	0.4612	0.2127	0.2042	0.4784	0.2289	0.2287	0.4286	0.1837	0.2421
PJLIML	0.6746	0.4551	0.0958	0.7393	0.5465	0.0559	0.8420	0.7089	0.0393	0.8572	0.7348	0.0172
PJFUL	0.6771	0.4585	0.0955	0.7406	0.5486	0.0559	0.8415	0.7082	0.0393	0.8572	0.7347	0.0172

Note: OLIML = “oracle-limited information maximum likelihood (LIML)”; NLIML = “naive-LIML”; OFUL = “oracle-FUL^17^”; NFUL = “naive-FUL”; PTSLS = “Penalized two-stage least square”^12^; proposed estimators: PLIML = “Penalized LIML”; PFUL = “Penalized FUL”; PJTSLS = “Penalized jackknife two-stage least square”; PJLIML = “Penalized jackknife-LIML”; PJFUL = “Penalized jackknife-FUL.” We report the median bias, median squared error (MSE) and average standard error (SE). The SEs of PTSLS, PLIML, PFUL, PJTSLS, PJLIML, and PJFUL are obtained by bootstrapping.

**Table 2b:** Estimation results of the estimators for *L* = 30 and *r* = 9 with heteroscedastic errors.

	*n* = 200			*n* = 500			*n* = 1000			*n* = 5000		
Estimators	Bias	MSE	SE	Bias	MSE	SE	Bias	MSE	SE	Bias	MSE	SE
$\sigma_{\mu e}=0.30$												
$\vartheta^{2}=8$												
OLIML	1.0657	1.1357	29.378	1.0373	1.0760	5.5995	1.1804	1.3940	102.12	1.0134	1.0269	21.336
NLIML	26.932	725.32	1313.2	45.216	2044.5	1023.7	59.681	3561.8	1483.5	134.10	17982	2562.8
OFUL	0.8484	0.7198	0.8153	0.7933	0.6293	0.9186	0.9289	0.8629	0.9093	0.7950	0.6320	1.3244
NFUL	16.807	282.46	10.038	27.524	757.57	14.813	37.084	1375.2	22.314	84.658	7166.9	43.856
PTSLS	0.9382	0.8803	0.4073	0.9293	0.8637	0.4410	0.9573	0.9164	0.4322	0.9165	0.8401	0.5669
PLIML	0.6432	0.4137	0.2081	0.5735	0.3289	0.1044	0.5559	0.3090	0.0656	0.5401	0.2917	0.0262
PFUL	0.6462	0.4176	0.2072		0.5752	0.3308	0.1041	0.5560	0.3092	0.0655	0.5402	0.2918	0.0262
PJTSLS	0.3902	0.1523	0.3749	0.3595	0.1292	0.3971	0.3926	0.1541	0.3948	0.3825	0.1463	0.6438
PJLIML	0.5801	0.3365	0.1553	0.5611	0.3149	0.0930	0.5518	0.3044	0.0624	0.5399	0.2915	0.0261
PJFUL	0.5805	0.3370	0.1550	0.5607	0.3144	0.0931	0.5518	0.3045	0.0624	0.5399	0.2915	0.0261
$\vartheta^{2}=64$												
OLIML	0.2971	0.0882	0.3000	0.2711	0.0735	0.2860	0.2590	0.0671	0.2908	0.2714	0.0737	0.4922
NLIML	10.296	106.00	4.8698	16.099	259.17	6.2305	22.444	503.74	7.0819	49.915	2491.5	16.011
OFUL	0.2898	0.0840	0.2903	0.2686	0.0721	0.2780	0.2499	0.0624	0.2837	0.2702	0.0730	0.4608
NFUL	9.6628	93.370	3.2726	15.185	230.58	4.7904	21.185	448.82	6.0105	47.083	2216.8	11.782
PTSLS	0.7797	0.6080	0.3264	0.7809	0.6099	0.3256	0.7821	0.6117	0.3637	0.7355	0.5409	0.5350
PLIML	0.6633	0.4400	0.2250	0.5874	0.3450	0.1130	0.5679	0.3225	0.0726	0.5403	0.2919	0.0277
PFUL	0.6659	0.4434	0.2246	0.5895	0.3475	0.1127	0.5671	0.3216	0.0725	0.5400	0.2916	0.0277
PJTSLS	0.3208	0.1029	0.2489	0.4256	0.1811	0.2456	0.4104	0.1684	0.2568	0.3484	0.1214	0.2595
PJLIML	0.5414	0.2932	0.1585	0.5614	0.3152	0.0944	0.5610	0.3147	0.0644	0.5397	0.2913	0.0277
PJFUL	0.5450	0.2970	0.1577	0.5595	0.3130	0.0943	0.5607	0.3144	0.0644	0.5396	0.2911	0.0276
$\sigma_{\mu e}=0.60$												
$\vartheta^{2}=8$												
OLIML	0.9229	0.8517	21.410	0.9494	0.9014	21.932	0.9962	0.9924	21.205	0.8760	0.7674	32.850
NLIML	29.745	884.77	328.80	47.804	2285.3	949.75	61.985	3842.1	1059.8	137.6	18921	2771.1
OFUL	0.6827	0.4661	0.5818	0.7156	0.5120	0.9403	0.7115	0.5062	0.8807	0.6242	0.3896	0.8332
NFUL	14.940	223.22	8.0492	23.498	552.17	14.429	30.905	955.13	17.739	71.287	5081.8	39.091
PTSLS	1.1169	1.2474	0.2376	1.1229	1.2610	0.3670	1.1413	1.3027	0.3904	1.1228	1.2608	0.2941
PLIML	1.0147	1.0297	0.1204	0.9931	0.9862	0.0727	1.0341	1.0694	0.0418	1.0328	1.0666	0.0183
PFUL	1.0132	1.0267	0.1201	0.9923	0.9847	0.0727	1.0340	1.0692	0.0420	1.0328	1.0666	0.0183
PJTSLS	0.4644	0.2156	0.3619	0.5384	0.2899	0.7924	0.5649	0.3191	0.7927	0.4526	0.2048	0.7395
PJLIML	0.9693	0.9396	0.0981	0.9806	0.9616	0.0713	1.0304	1.0618	0.0411	1.0327	1.0665	0.0182
PJFUL	0.9754	0.9514	0.0981	0.9818	0.9638	0.0711	1.0312	1.0634	0.0411	1.0327	1.0664	0.0182
$\vartheta^{2}=64$												
OLIML	0.2583	0.0667	0.3674	0.2760	0.0762	0.6012	0.2830	0.0801	0.7474	0.2437	0.0594	0.7249
NLIML	12.715	161.67	40.977	18.000	324.01	16.607	24.471	598.83	49.728	49.801	2480.1	24.572
OFUL	0.2590	0.0671	0.3213	0.2702	0.0730	0.4851	0.2612	0.0682	0.4400	0.2348	0.0552	0.4709
NFUL	11.086	122.91	5.0926	16.323	266.46	4.2491	22.053	486.32	5.5862	45.612	2080.5	11.663
PTSLS	0.8463	0.7162	0.2453	0.8767	0.7686	0.3353	0.8692	0.7555	0.3076	0.8604	0.7403	0.3339
PLIML	0.9084	0.8252	0.1519	0.9571	0.9160	0.0746	1.0128	1.0257	0.0412	1.0285	1.0577	0.0162
PFUL	0.9068	0.8223	0.1508	0.9576	0.9170	0.0756	1.0125	1.0251	0.0415	1.0284	1.0576	0.0162
PJTSLS	0.3469	0.1203	0.2283	0.5366	0.2879	0.2844	0.5294	0.2803	0.2832	0.4685	0.2195	0.2366
PJLIML	0.8288	0.6868	0.1013	0.9415	0.8865	0.0685	1.0085	1.0172	0.0379	1.0281	1.0569	0.0161
PJFUL	0.8323	0.6927	0.1014	0.9425	0.8884	0.0682	1.0089	1.0179	0.0380	1.0280	1.0568	0.0161

Note: OLIML = “oracle-limited information maximum likelihood (LIML)”; NLIML = “naive-LIML”; OFUL = “oracle-FUL^17^”; NFUL = “naive-FUL”; PTSLS = “Penalized two-stage least square”^12^; proposed estimators: PLIML = “Penalized LIML”; PFUL = “Penalized FUL”; PJTSLS = “Penalized jackknife two-stage least square”; PJLIML = “Penalized jackknife-LIML”; PJFUL = “Penalized jackknife-FUL”. We report the median bias, median squared error (MSE) and average standard error (SE). The SEs of PTSLS, PLIML, PFUL, PJTSLS, PJLIML, and PJFUL are obtained by bootstrapping.

**Table 2c:** Estimation results of the estimators for *L* = 60 and *r* = 18 with heteroscedastic errors.

	*n* = 200			*n* = 500			*n* = 1000			*n* = 5000		
Estimators	Bias	MSE	SE	Bias	MSE	SE	Bias	MSE	SE	Bias	MSE	SE
$\sigma_{\mu e}=0.30$												
$\vartheta^{2}=8$												
OLIML	1.6016	2.5650	33.191	1.5904	2.5293	56.544	1.6537	2.7349	30.563	1.5317	2.3462	86.343
NLIML	40.025	1602.0	7483.1	60.407	3649.0	905.24	94.479	8926.3	15105	204.53	41831	8865.1
OFUL	1.3027	1.6971	1.1471	1.2720	1.6181	1.8644	1.2709	1.6152	1.8996	1.2555	1.5763	1.9022
NFUL	23.481	551.36	13.090	36.699	1346.8	17.491	53.874	2902.4	28.263	119.60	14303	60.579
PTSLS	1.1421	1.3043	0.3571	1.2088	1.4612	0.4339	1.1716	1.3727	0.4248	1.1980	1.4352	0.4407
PLIML	0.9762	0.9530	0.2991	0.8969	0.8044	0.1304	0.8613	0.7419	0.0836	0.8338	0.6952	0.0359
PFUL	0.9752	0.9511	0.2989	0.8972	0.8050	0.1305	0.8612	0.7417	0.0836	0.8339	0.6953	0.0359
PJTSLS	0.7287	0.5311	0.3083	0.4104	0.1684	0.5862	0.3954	0.1563	0.6243	0.3938	0.1551	0.6229
PJLIML	0.7982	0.6371	0.2007	0.8564	0.7334	0.1214	0.8513	0.7247	0.0816	0.8331	0.6941	0.0358
PJFUL	0.8045	0.6472	0.2004	0.8582	0.7365	0.1217	0.8520	0.7258	0.0817	0.8330	0.6939	0.0358
$\vartheta^{2}=64$												
OLIML	0.4873	0.2374	0.3910	0.4340	0.1883	0.8118	0.4319	0.1866	1.8343	0.4221	0.1782	0.9424
NLIML	15.229	231.91	16.446	22.927	525.64	11.967	32.974	1087.3	11.663	69.909	4887.3	28.533
OFUL	0.4769	0.2275	0.3748	0.4177	0.1745	0.6988	0.4179	0.1746	0.7342	0.4059	0.1648	0.7547
NFUL	14.138	199.89	5.1201	21.502	462.35	5.5510	30.875	953.27	7.3416	65.820	4332.3	17.888
PTSLS	1.1797	1.3916	0.3259	1.1765	1.3842	0.3187	1.1285	1.2735	0.3048	1.1331	1.2839	0.3169
PLIML	1.0752	1.1561	0.2953	0.9354	0.8749	0.1408	0.8774	0.7698	0.0877	0.8359	0.6987	0.0353
PFUL	1.0792	1.1647	0.2955	0.9359	0.8760	0.1413	0.8770	0.7691	0.0874	0.8361	0.6991	0.0353
PJTSLS	0.2135	0.0456	0.2530	0.4401	0.1937	0.3000	0.5047	0.2547	0.2808	0.4451	0.1981	0.3079
PJLIML	0.7295	0.5322	0.1900	0.8569	0.7343	0.1238	0.8577	0.7357	0.0828	0.8348	0.6969	0.0352
PJFUL	0.7394	0.5467	0.1900	0.8588	0.7375	0.1234	0.8608	0.7410	0.0833	0.8352	0.6975	0.0352
$\sigma_{\mu e}=0.60$												
$\vartheta^{2}=8$												
OLIML	1.3394	1.7940	100.59	1.0993	1.2084	24.999	1.2648	1.5998	20.176	1.1392	1.2978	20.851
NLIML	41.294	1705.2	2334.6	61.263	3753.1	6260.8	87.847	7717.3	883.76	185.07	34251	3615.1
OFUL	0.9649	0.9311	0.5840	0.8645	0.7473	0.8525	0.8870	0.7867	0.9652	0.8842	0.7819	0.9004
NFUL	18.724	350.60	10.043	26.328	693.16	17.553	36.237	1313.2	25.606	84.375	7119.1	59.068
PTSLS	1.3352	1.7827	0.1456	1.2760	1.6283	0.1819	1.2794	1.6368	0.1924	1.2817	1.6428	0.1737
PLIML	1.2824	1.6445	0.1210	1.2236	1.4973	0.0532	1.2208	1.4903	0.0372	1.2157	1.4780	0.0142
PFUL	1.2802	1.6388	0.1210	1.2229	1.4954	0.0529	1.2212	1.4914	0.0372	1.2157	1.4780	0.0142
PJTSLS	0.8560	0.7328	0.2903	0.5758	0.3315	0.6479	0.5809	0.3375	0.7322	0.4955	0.2456	0.7128
PJLIML	1.1412	1.3023	0.1325	1.2011	1.4426	0.0526	1.2153	1.4770	0.0363	1.2155	1.4773	0.0142
PJFUL	1.1495	1.3214	0.1328	1.2022	1.4452	0.0527	1.2149	1.4760	0.0362	1.2155	1.4774	0.0142
$\vartheta^{2}=64$												
OLIML	0.4232	0.1791	3.7552	0.4018	0.1615	8.2432	0.3717	0.1382	2.1096	0.3776	0.1425	3.5872
NLIML	17.365	301.54	569.79	24.832	616.65	482.16	33.854	1146.1	39.008	74.350	5528.1	444.66
OFUL	0.3933	0.1547	0.4937	0.3811	0.1453	0.6661	0.3592	0.1290	0.6280	0.3473	0.1206	0.6036
NFUL	14.500	210.26	6.5774	21.169	448.13	6.6786	29.408	864.85	8.1396	64.119	4111.2	17.025
PTSLS	1.1870	1.4089	0.1608	1.1306	1.2782	0.2104	1.1304	1.2779	0.2018	1.1229	1.2608	0.1732
PLIML	1.1965	1.4316	0.1406	1.1926	1.4224	0.0653	1.2012	1.4429	0.0399	1.2124	1.4699	0.0152
PFUL	1.1952	1.4286	0.1403	1.1931	1.4234	0.0649	1.2006	1.4414	0.0400	1.2123	1.4697	0.0152
PJTSLS	0.2970	0.0882	0.2344	0.3406	0.1160	0.4128	0.4662	0.2174	0.3854	0.4458	0.1987	0.3836
PJLIML	1.0031	1.0062	0.1201	1.1597	1.3448	0.0596	1.1902	1.4165	0.0375	1.2118	1.4684	0.0151
PJFUL	1.0109	1.0219	0.1199	1.1616	1.3493	0.0597	1.1901	1.4163	0.0377	1.2121	1.4693	0.0151

Note: OLIML = “oracle-limited information maximum likelihood (LIML)”; NLIML = “naive-LIML”; OFUL = “oracle-FUL^17^”; NFUL = “naive-FUL”; PTSLS = “Penalized two-stage least square”^12^; proposed estimators: PLIML = “Penalized LIML”; PFUL = “Penalized FUL”; PJTSLS = “Penalized jackknife two-stage least square”; PJLIML = “Penalized jackknife-LIML”; PJFUL = “Penalized jackknife-FUL”. We report the median bias, median squared error (MSE) and average standard error (SE). The SEs of PTSLS, PLIML, PFUL, PJTSLS, PJLIML, and PJFUL are obtained by bootstrapping.

In Tables 3a–3c, the values of bias, MSE and mean standard errors are lower than those in the heteroscedastic case. Tables 3a–3c provide interesting findings for different cases. For example, when $\sigma_{e\mu }=0.30$ and $\vartheta^{2}=8$ , the causal effect estimates of PJLIML and PJFUL perform efficiently and have substantially lower bias, MSE and standard errors than those of the other methods do. This is the benefit of the PJLIML and PJFUL methods under many (weak) instruments. On the other hand, when the instruments are not very weak $\left(\vartheta^{2}=64\right)$ and $\sigma_{e\mu }=0.30$ , PJTSLS seems to perform better than the other methods do. When $\vartheta^{2}=8$ and $\sigma_{e\mu }=0.30$ , OLIML and OFUL have higher MSEs. This is because both the LIML and FUL estimators are inconsistent and exhibit greater dispersion, particularly for LIML, due to the “moments problem” under conditions of many (weak) instruments and heteroskedasticity. However, even under homoscedasticity, the issue of many weak instruments remains. With many (weak) instruments, ${P_{Z}}_{ii}$ does not shrink to zero, causing inconsistency. When $\vartheta^{2}=64$ , the OLIML and OFUL estimators perform better than the other methods do, as expected. The performance of PTSLS and PJTSLS is superior to that of other penalized methods when the instruments are slightly strong and the degree of endogeneity is high (Tables 3a and 3b); when L=60 (Table 3c), the bias, MSE and mean standard error of PJLIML and PJFUL are lower than those of PTSLS. The median bias, MSE, and mean standard error values generally decrease as *n* increases, but this is not the case for all estimators, and the pattern is not consistent. The parameter ${\psi_{0}}_{j}$ varies with the sample size and number of instruments and is not constant, as shown in Tables 2 and 3. However, in Model I, we fix the value of ${\psi_{0}}_{j}$ , and it can be seen in Table 1 that the MSE decreases when the sample size increases, and the performance of the estimators improves.

**Table 3a.** Estimation results of the estimators for *L* = 15 and *r* = 5 with homoscedastic errors.

	*n* = 200			*n* = 500			*n* = 1000			*n* = 5000		
Estimators	Bias	MSE	SE	Bias	MSE	SE	Bias	MSE	SE	Bias	MSE	SE
$\sigma_{\mu e}=0.30$												
$\vartheta^{2}=8$												
OLIML	0.4436	0.1968	5.8441	0.4509	0.2033	69.011	0.4449	0.1979	4.7034	0.4007	0.1605	4.3224
NLIML	20.316	412.74	144.41	30.738	944.83	950.67	44.039	1939.5	781.57	96.750	9360.7	901.99
OFUL	0.3634	0.1320	0.4829	0.3766	0.1418	0.5064	0.3589	0.1288	0.5807	0.3375	0.1139	0.6042
NFUL	13.202	174.29	6.8089	20.268	410.80	11.210	28.916	836.16	13.273	64.967	4220.7	24.550
PTSLS	0.5113	0.2615	0.5526	0.5278	0.2786	0.6827	0.5114	0.2616	1.6302	0.4876	0.2378	3.5702
PLIML	0.2469	0.0610	0.1292	0.2202	0.0485	0.0683	0.2083	0.0434	0.0478	0.2027	0.0411	0.0191
PFUL	0.2471	0.0611	0.1281	0.2197	0.0483	0.0681	0.2081	0.0433	0.0465	0.2027	0.0411	0.0192
PJTSLS	0.2289	0.0524	0.3156	0.2317	0.0537	0.3303	0.2086	0.0435	0.4501	0.2360	0.0557	0.4323
PJLIML	0.2228	0.0497	0.0808	0.2145	0.0460	0.0475	0.2044	0.0418	0.0318	0.2020	0.0408	0.0132
PJFUL	0.2225	0.0495	0.0802	0.2141	0.0458	0.0474	0.2045	0.0418	0.0317	0.2020	0.0408	0.0131
$\vartheta^{2}=64$												
OLIML	0.1141	0.0130	0.1661	0.1086	0.0118	0.1877	0.1134	0.0128	0.1811	0.1179	0.0139	0.1840
NLIML	7.0139	49.194	2.3171	11.161	124.56	4.3601	15.532	241.25	5.0710	33.885	1148.2	8.4775
OFUL	0.1112	0.0124	0.1622	0.1062	0.0113	0.1778	0.1081	0.0117	0.1755	0.1146	0.0131	0.1776
NFUL	6.6533	44.266	1.9543	10.634	113.08	2.7209	14.827	219.85	3.7154	32.410	1050.41	6.8950
PTSLS	0.3778	0.1427	0.3073	0.3764	0.1417	0.2946	0.3711	0.1377	0.2710	0.3749	0.1405	0.3400
PLIML	0.2853	0.0814	0.1473	0.2341	0.0548	0.0589	0.2158	0.0466	0.0348	0.2020	0.0408	0.0142
PFUL	0.2849	0.0812	0.1459	0.2340	0.0547	0.0608	0.2158	0.0466	0.0345	0.2019	0.0408	0.0149
PJTSLS	0.1762	0.0311	0.1526	0.1820	0.0331	0.1481	0.1738	0.0302	0.1500	0.1768	0.0313	0.1578
PJLIML	0.2351	0.0553	0.0844	0.2220	0.0493	0.0462	0.2119	0.0449	0.0327	0.2019	0.0408	0.0139
PJFUL	0.2359	0.0556	0.0840	0.2229	0.0497	0.0458	0.2108	0.0444	0.0328	0.2016	0.0406	0.0138
$\sigma_{\mu e}=0.60$												
$\vartheta^{2}=8$												
OLIML	0.4194	0.1759	8.5442	0.4270	0.1824	3.8392	0.4117	0.1695	4.2641	0.4384	0.1922	40.637
NLIML	20.191	407.67	952.88	33.076	1094.0	283.59	45.079	2032.1	41204	100.78	10156	2500.5
OFUL	0.3196	0.1021	0.5376	0.3263	0.1065	0.4605	0.3065	0.0939	0.4999	0.3483	0.1213	0.5414
NFUL	13.132	172.46	5.5014	20.791	432.27	11.057	29.647	878.97	12.308	65.035	4229.5	30.933
PTSLS	0.7291	0.5316	0.8992	0.7091	0.5029	0.6572	0.7127	0.5079	1.3109	0.7258	0.5268	3.8978
PLIML	0.6237	0.3890	0.1149	0.6099	0.3719	0.0595	0.6038	0.3645	0.0378	0.6020	0.3625	0.0188
PFUL	0.6221	0.3870	0.1132	0.6104	0.3726	0.0590	0.6036	0.3643	0.0378	0.6020	0.3624	0.0178
PJTSLS	0.3963	0.1570	0.6033	0.3662	0.1341	0.3480	0.3194	0.1020	0.5168	0.2878	0.0828	0.5566
PJLIML	0.6056	0.3668	0.0636	0.6043	0.3652	0.0388	0.6018	0.3622	0.0255	0.6010	0.3612	0.0116
PJFUL	0.6053	0.3664	0.0635	0.6045	0.3654	0.0387	0.6019	0.3623	0.0256	0.6008	0.3610	0.0116
$\vartheta^{2}=64$												
OLIML	0.1128	0.0127	0.1696	0.1075	0.0116	0.1797	0.1111	0.0124	0.1677	0.1095	0.0120	0.1728
NLIML	8.3114	69.080	3.7003	12.204	148.94	5.0255	16.219	263.05	7.0103	35.218	1240.3	10.481
OFUL	0.1078	0.0116	0.1643	0.1044	0.0109	0.1697	0.1088	0.0118	0.1591	0.1058	0.0112	0.1635
NFUL	7.7859	60.620	2.6061	11.555	133.53	2.8426	15.422	237.85	3.5974	33.635	1131.3	7.8079
PTSLS	0.4535	0.2057	0.2926	0.4595	0.2111	0.3092	0.4599	0.2115	0.4923	0.4363	0.1904	0.5521
PLIML	0.5259	0.2766	0.1423	0.5708	0.3258	0.0551	0.5881	0.3458	0.0432	0.5972	0.3566	0.0163
PFUL	0.5303	0.2812	0.1412	0.5709	0.3260	0.0554	0.5874	0.3450	0.0453	0.5971	0.3566	0.0162
PJTSLS	0.2961	0.0877	0.1514	0.2994	0.0896	0.1350	0.2849	0.0812	0.1262	0.2475	0.0613	0.1420
PJLIML	0.4935	0.2435	0.0766	0.5638	0.3179	0.0388	0.5864	0.3439	0.0262	0.5969	0.3562	0.0118
PJFUL	0.4960	0.2460	0.0764	0.5639	0.3180	0.0388	0.5853	0.3426	0.0264	0.5970	0.3564	0.0118

Note: OLIML = “oracle-limited information maximum likelihood (LIML)”; NLIML = “naive-LIML”; OFUL = “oracle-FUL^17^”; NFUL = “naive-FUL”; PTSLS = “Penalized two-stage least square”^12^; proposed estimators: PLIML = “Penalized LIML”; PFUL = “Penalized FUL”; PJTSLS = “Penalized jackknife two-stage least square”; PJLIML = “Penalized jackknife-LIML”; PJFUL = “Penalized jackknife-FUL”. We report the median bias, median squared error (MSE) and average standard error (SE). The SEs of PTSLS, PLIML, PFUL, PJTSLS, PJLIML, and PJFUL are obtained by bootstrapping.

**Table 3b:** Estimation results of the estimators for *L* = 30 and *r* = 9 with homoscedastic errors.

	*n* = 200			*n* = 500			*n* = 1000			*n* = 5000		
Estimators	Bias	MSE	SE	Bias	MSE	SE	Bias	MSE	SE	Bias	MSE	SE
$\sigma_{\mu e}=0.30$												
$\vartheta^{2}=8$												
OLIML	0.5471	0.2994	21.549	0.4703	0.2211	42.657	0.5558	0.3089	9.8970	0.5340	0.2852	152.65
NLIML	25.830	667.21	13907	42.400	1797.7	366.75	61.821	3821.9	1791.4	136.54	18642	3532.7
OFUL	0.4401	0.1937	0.7109	0.3925	0.1541	0.5172	0.4612	0.2127	0.7620	0.4316	0.1863	0.7321
NFUL	17.238	297.14	8.8823	27.841	775.10	15.164	39.229	1538.9	18.265	85.414	7295.6	43.086
PTSLS	0.4392	0.1929	0.3451	0.4551	0.2071	0.2727	0.4400	0.1936	0.3315	0.4472	0.2000	0.9151
PLIML	0.2664	0.0710	0.1032	0.2242	0.0503	0.0581	0.2101	0.0441	0.0344	0.2035	0.0414	0.0151
PFUL	0.2643	0.0698	0.1024	0.2237	0.0501	0.0581	0.2100	0.0441	0.0344	0.2036	0.0414	0.0154
PJTSLS	0.3820	0.1459	0.3949	0.2533	0.0641	0.2463	0.2735	0.0748	0.4134	0.2993	0.0896	0.4104
PJLIML	0.2209	0.0488	0.0813	0.2137	0.0457	0.0497	0.2068	0.0428	0.0332	0.2032	0.0413	0.0136
PJFUL	0.2211	0.0489	0.0812	0.2146	0.0460	0.0497	0.2070	0.0428	0.0332	0.2033	0.0413	0.0136
$\vartheta^{2}=64$												
OLIML	0.1233	0.0152	0.1733	0.1274	0.0162	0.1597	0.1164	0.0135	0.2028	0.1242	0.0154	0.2245
NLIML	9.9734	99.469	5.2307	15.963	254.83	4.5340	21.762	473.58	6.8518	48.991	2400.1	15.711
OFUL	0.1212	0.0147	0.1670	0.1250	0.0156	0.1569	0.1123	0.0126	0.1927	0.1178	0.0139	0.2052
NFUL	9.4271	88.871	3.0074	15.119	228.58	4.0042	20.697	428.37	5.0801	46.527	2164.8	10.885
PTSLS	0.3997	0.1597	0.2007	0.3874	0.1501	0.2013	0.4029	0.1623	0.2462	0.3909	0.1528	0.1774
PLIML	0.3128	0.0978	0.1345	0.2440	0.0595	0.0661	0.2227	0.0496	0.0330	0.2043	0.0417	0.0136
PFUL	0.3127	0.0978	0.1334	0.2432	0.0591	0.0661	0.2226	0.0496	0.0333	0.2042	0.0417	0.0136
PJTSLS	0.0975	0.0095	0.1397	0.1104	0.0122	0.1392	0.1144	0.0131	0.1382	0.1147	0.0132	0.1576
PJLIML	0.2099	0.0441	0.0849	0.2214	0.0490	0.0508	0.2166	0.0469	0.0315	0.2040	0.0416	0.0136
PJFUL	0.2112	0.0446	0.0846	0.2234	0.0499	0.0507	0.2168	0.0470	0.0313	0.2039	0.0416	0.0136
$\sigma_{\mu e}=0.60$												
$\vartheta^{2}=8$												
OLIML	0.5054	0.2554	13.595	0.4778	0.2283	6.8080	0.4441	0.1973	41.860	0.4429	0.1962	4.0026
NLIML	30.265	915.95	258.86	42.381	1796.2	553.95	61.359	3764.9	1967.5	127.46	16247	861.31
OFUL	0.3960	0.1568	0.6276	0.3826	0.1464	0.4761	0.3591	0.1290	0.6304	0.3540	0.1253	0.6028
NFUL	17.565	308.53	8.7924	27.788	772.17	15.538	39.767	1581.4	18.695	85.616	7330.1	39.838
PTSLS	0.7063	0.4989	0.3245	0.7283	0.5304	0.2200	0.7177	0.5151	0.2609	0.7190	0.5170	0.2651
PLIML	0.6290	0.3957	0.0794	0.6105	0.3727	0.0466	0.6054	0.3665	0.0263	0.6008	0.3610	0.0119
PFUL	0.6301	0.3970	0.0794	0.6104	0.3726	0.0465	0.6054	0.3666	0.0264	0.6010	0.3612	0.0119
PJTSLS	0.3523	0.1241	0.4747	0.3077	0.0947	0.2607	0.2928	0.0858	0.4905	0.2780	0.0773	0.5198
PJLIML	0.5906	0.3488	0.0679	0.6024	0.3629	0.0403	0.6034	0.3641	0.0258	0.6007	0.3608	0.0119
PJFUL	0.5933	0.3519	0.0679	0.6030	0.3636	0.0402	0.6034	0.3641	0.0258	0.6006	0.3607	0.0119
$\vartheta^{2}=64$												
OLIML	0.1227	0.0151	0.1715	0.1236	0.0153	0.1413	0.1070	0.0114	0.1804	0.1061	0.0113	0.1886
NLIML	11.490	132.01	9.4369	17.348	300.94	6.0294	23.708	562.05	8.0123	50.220	2522.1	16.159
OFUL	0.1201	0.0144	0.1650	0.1201	0.0144	0.1337	0.1049	0.0110	0.1709	0.0999	0.0100	0.1742
NFUL	10.669	113.83	3.3918	16.262	264.46	3.7447	22.359	499.91	5.5266	47.644	2270.0	11.591
PTSLS	0.5005	0.2505	0.1701	0.5087	0.2588	0.1712	0.5064	0.2564	0.1109	0.5046	0.2546	0.1183
PLIML	0.5440	0.2959	0.1169	0.5764	0.3323	0.0509	0.5901	0.3483	0.0259	0.5975	0.3570	0.0110
PFUL	0.5455	0.2976	0.1165	0.5748	0.3304	0.0508	0.5900	0.3481	0.0260	0.5975	0.3570	0.0110
PJTSLS	0.1591	0.0253	0.1419	0.2473	0.0612	0.1299	0.2417	0.0584	0.1426	0.2064	0.0426	0.1590
PJLIML	0.4621	0.2136	0.0781	0.5593	0.3128	0.0461	0.5849	0.3421	0.0257	0.5973	0.3568	0.0110
PJFUL	0.4663	0.2174	0.0777	0.5605	0.3142	0.0461	0.5860	0.3434	0.0257	0.5974	0.3569	0.0110

Note: OLIML = “oracle-limited information maximum likelihood (LIML)”; NLIML = “naive-LIML”; OFUL = “oracle-FUL^17^”; NFUL = “naive-FUL”; PTSLS = “Penalized two-stage least square”^12^; proposed estimators: PLIML = “Penalized LIML”; PFUL = “Penalized FUL”; PJTSLS = “Penalized jackknife two-stage least square”; PJLIML = “Penalized jackknife-LIML”; PJFUL = “Penalized jackknife-FUL”. We report the median bias, median squared error (MSE) and average standard error (SE). The SEs of PTSLS, PLIML, PFUL, PJTSLS, PJLIML, and PJFUL are obtained by bootstrapping.

**Table 3c:** Estimation results of the estimators for *L* = 60 and *r* = 18 with homoscedastic errors.

	*n* = 200			*n* = 500			*n* = 1000			*n* = 5000		
Estimators	Bias	MSE	SE	Bias	MSE	SE	Bias	MSE	SE	Bias	MSE	SE
$\sigma_{\mu e}=0.30$												
$\vartheta^{2}=8$												
OLIML	0.6319	0.3993	28.18	0.6951	0.4832	34.688	0.5969	0.3563	53.133	0.6515	0.4245	4.1784
NLIML	40.163	1613.0	1250.5	62.666	3927.1	6285.9	85.420	7296.7	1573.3	201.00	40403	4200.7
OFUL	0.5311	0.2820	0.5342	0.5365	0.2878	0.9070	0.5135	0.2637	0.8943	0.5622	0.3161	0.8724
NFUL	24.533	601.88	13.192	38.753	1501.8	19.718	55.412	3070.5	23.418	125.53	15758	61.155
PTSLS	0.3828	0.1465	0.1833	0.3866	0.1495	0.2255	0.4128	0.1704	0.2115	0.3904	0.1524	0.2196
PLIML	0.3001	0.0901	0.1508	0.2332	0.0544	0.0562	0.2176	0.0474	0.0335	0.2025	0.0410	0.0132
PFUL	0.2981	0.0889	0.1504	0.2325	0.0541	0.0562	0.2176	0.0474	0.0334	0.2025	0.0410	0.0132
PJTSLS	0.8082	0.6532	0.1713	0.5515	0.3042	0.3493	0.4562	0.2081	0.3339	0.4811	0.2314	0.3373
PJLIML	0.1768	0.0313	0.1021	0.2105	0.0443	0.0496	0.2098	0.0440	0.0320	0.2021	0.0409	0.0132
PJFUL	0.1817	0.0330	0.1022	0.2115	0.0448	0.0498	0.2107	0.0444	0.0318	0.2021	0.0409	0.0132
$\vartheta^{2}=64$												
OLIML	0.1679	0.0282	0.1949	0.1609	0.0259	0.8578	0.1369	0.0188	0.2990	0.1539	0.0237	0.2949
NLIML	14.157	200.41	7.8704	21.546	464.24	6.1868	30.964	958.77	11.220	69.339	4807.9	18.756
OFUL	0.1656	0.0274	0.1805	0.1556	0.0242	0.2692	0.1352	0.0183	0.2536	0.1483	0.0220	0.2394
NFUL	13.395	179.43	4.1468	20.486	419.69	4.7906	29.357	861.81	7.6277	65.819	4332.2	14.654
PTSLS	0.4573	0.2091	0.1622	0.4589	0.2106	0.1626	0.4382	0.1920	0.1560	0.4426	0.1959	0.1566
PLIML	0.3898	0.1519	0.1457	0.2814	0.0792	0.0603	0.2381	0.0567	0.0343	0.2068	0.0428	0.0140
PFUL	0.3886	0.1510	0.1458	0.2826	0.0799	0.0603	0.2377	0.0565	0.0344	0.2067	0.0427	0.0140
PJTSLS	0.3102	0.0962	0.1306	0.1050	0.0110	0.1436	0.0909	0.0083	0.1430	0.0946	0.0090	0.1441
PJLIML	0.1473	0.0217	0.0912	0.2254	0.0508	0.0484	0.2214	0.0490	0.0314	0.2060	0.0424	0.0139
PJFUL	0.1507	0.0227	0.0908	0.2270	0.0515	0.0476	0.2211	0.0489	0.0315	0.2060	0.0424	0.0139
$\sigma_{\mu e}=0.60$												
$\vartheta^{2}=8$												
OLIML	0.6402	0.4099	6.7102	0.6392	0.4086	3.9629	0.5901	0.3482	44.322	0.5362	0.2875	18.216
NLIML	41.121	1690.9	718.78	61.199	3745.3	1228.4	84.972	7220.3	2471.9	193.66	37504	7640.6
OFUL	0.5035	0.2536	0.5007	0.5216	0.2721	0.7583	0.4657	0.2169	0.7513	0.4348	0.1891	0.7327
NFUL	24.840	617.05	14.087	40.036	1602.9	17.955	55.410	3070.3	25.020	124.36	15466	52.479
PTSLS	0.7009	0.4913	0.1480	0.7297	0.5324	0.1628	0.7250	0.5256	0.1838	0.7155	0.5119	0.1740
PLIML	0.6542	0.4280	0.1223	0.6238	0.3891	0.0440	0.6118	0.3744	0.0284	0.6025	0.3630	0.0115
PFUL	0.6523	0.4255	0.1221	0.6227	0.3878	0.0440	0.6128	0.3755	0.0283	0.6025	0.3630	0.0116
PJTSLS	0.8082	0.6531	0.1969	0.4472	0.2000	0.3772	0.3281	0.1077	0.4369	0.3393	0.1151	0.4227
PJLIML	0.5395	0.2911	0.1045	0.6007	0.3608	0.0412	0.6053	0.3664	0.0275	0.6023	0.3628	0.0115
PJFUL	0.5488	0.3012	0.1045	0.6016	0.3619	0.0411	0.6057	0.3669	0.0275	0.6022	0.3627	0.0115
$\vartheta^{2}=64$												
OLIML	0.1449	0.0210	0.2657	0.1272	0.0162	0.2634	0.1436	0.0206	0.2292	0.1358	0.0184	0.2270
NLIML	14.864	220.95	7.2045	22.758	517.90	9.6079	32.531	1058.3	10.686	70.598	4984.1	20.710
OFUL	0.1401	0.0196	0.2385	0.1194	0.0143	0.2277	0.1453	0.0211	0.2131	0.1304	0.0170	0.2126
NFUL	14.007	196.21	4.1371	21.569	465.21	5.3666	30.715	943.41	7.5753	66.835	4466.9	15.559
PTSLS	0.6098	0.3718	0.1415	0.5981	0.3577	0.1344	0.6034	0.3641	0.1216	0.5933	0.3520	0.1158
PLIML	0.6078	0.3694	0.1047	0.6020	0.3624	0.0503	0.6002	0.3603	0.0288	0.5987	0.3584	0.0119
PFUL	0.6069	0.3684	0.1041	0.6021	0.3625	0.0500	0.6002	0.3603	0.0290	0.5987	0.3584	0.0119
PJTSLS	0.2468	0.0609	0.1886	0.1439	0.0207	0.1755	0.1893	0.0359	0.1738	0.1800	0.0324	0.1763
PJLIML	0.4010	0.1608	0.0870	0.5552	0.3082	0.0438	0.5854	0.3427	0.0272	0.5982	0.3579	0.0118
PJFUL	0.4061	0.1649	0.0873	0.5574	0.3107	0.0437	0.5859	0.3433	0.0273	0.5983	0.3580	0.0118

Note: OLIML = “oracle-limited information maximum likelihood (LIML)”; NLIML = “naive-LIML”; OFUL = “oracle-FUL^17^”; NFUL = “naive-FUL”; PTSLS = “Penalized two-stage least square”^12^; proposed estimators: PLIML = “Penalized LIML”; PFUL = “Penalized FUL”; PJTSLS = “Penalized jackknife two-stage least square”; PJLIML = “Penalized jackknife-LIML”; PJFUL = “Penalized jackknife-FUL”. We report the median bias, median squared error (MSE) and average standard error (SE). The SEs of PTSLS, PLIML, PFUL, PJTSLS, PJLIML, and PJFUL are obtained by bootstrapping.

The results of OLIML and OFUL achieve better performances than the naive estimators because the oracle estimators accurately identify which instruments are valid and invalid. However, the naive estimators (NLIML and NFUL) assume that all the instruments are valid, and consequently, they have higher bias, MSE and mean standard error values than the other estimators do. Note that the proposed estimators do not use the information that one knows accurately which instruments are valid, whereas the TSLS, LIML and FUL estimators do. Examining the FUL- and LIML-type estimators reveals that FUL is less dispersed than LIML. The proposed estimators perform similar to the oracle estimators and sometimes perform even better. The LASSO-type jackknife IV estimators outperform the PTSLS estimator. In summary, these simulation results indicate that the PTSLS performs worse when the instruments are weak and the errors are heteroscedastic, so PJLIML and PJFUL may be helpful methods when many instruments are used. Moreover, PJTSLS performs well relative to all other estimators.

## 5. Analysis of body mass index, health-related quality of life and genetic markers

This analysis was conducted to perform an MR study in which we estimated the causal effect of BMI on the HRQLI using SNPs as instruments for BMI. The HRQLI is estimated via the health utility index mark 3 developed by Horsman et al.,^40^ which is a summary measure of several health attributes, such as vision, hearing and cognitive skills. A health utility score of 1 indicates “perfect health,” and a value of 0 represents a “dead” state. The health utility score can be negative, which represents a state “worse than death.”^41,42^ We use data from the Wisconsin Longitudinal Study (WLS),^5^ which includes American high school graduates from Wisconsin who have been tracked since 1957. According to the information provided by the WLS, genetic variants can explain different dimensions of the HRQLI (e.g. cognitive skills). Our analysis is limited to 1816 individuals who were genotyped in 2004. We remove individuals with more than 10% missing genotype data. We use 10 genetic variants (SNPs) as potential IVs that have been used in previous research either to explain various dimensions of HRQLI or as instruments explaining BMI. The SNPs used as potential instruments (APOE, CHRM2, GABBR2,5-HTR2A, ADIPOQ, DISCI, CYP11A1, BDNF, HFE and DRD2), along with the respective references for each SNP, are summarized in Table 4. In addition, the diseases/behavior associated with them as identified by WLS are also presented in Table 4. IVs may be invalid for various reasons, such as linkage disequilibrium, population stratification, and horizontal pleiotropy.^13,53^ The R code for the analysis of BMI, HRQLI and genetic variants is provided in the supplementary material.^6^

**Table 4.** Summary of the genetic instruments.

Instruments**^†^**	SNP ID*	Disease/Behavior	Authors
APOE	rs429358	Alzheimer's	^43^
CHRM2	rs2061174	Cognition	^44^
GABBR2	rs1435252	Nicotine Addiction	^45^
HTR2A	rs6314	Memory Performance	^46,47^
ADIPOQ	rs2241766	Diabetes II, Obesity	^48^
DISC1	rs821616	Cognitive Aging, Schizophrenia	Bischof and Park^49^
CYP11A1	rs8039957	Cognitive Aging	Bischof and Park^49^
BDNF	rs6265	Cognitive Aging, Memory, IQ	^44,50^
HFE	rs1799945	Alzheimer's, Obesity, Liver Disease	Määttä et al.^51^
DRD2	rs1800497	Nicotine/Alcohol Addiction	^44,52^

Note: ^†^APOE = “apolipoprotein E”; CHRM2 = “cholinergic muscarinic receptor 2”; GABAB2= “gamma-aminobutyric acid type B receptor subunit 2 gene”; HTR2A = “5-hydroxytryptamine (serotonin) receptor 2A”; ADIPOQ = “adiponectin”; DISC1= “disrupted-in-schizophrenia 1”; CYP11A1= “cholesterol side chain cleavage enzyme that catalyzes the initial and rate-limiting step of steroidogenesis”; BDNF = “brain-derived neurotrophic factor”; HFE = “human homeostatic iron regulator protein”; DRD2= “dopamine receptor D2 gene”. *“rsID” is a unique label used to identify a specific single nucleotide polymorphism (SNP).

The parameter of interest for estimating the causal effect of BMI on the HRQLI is $\beta_{0}$ in Model (2.1). The results of the estimated causal effect ( $\hat{\beta }$ ), standard errors, 95% confidence intervals and number of invalid IVs from the causal regression model using SNPs are given in Table 5. If we treat all instruments as valid, then the causal effects for the TSLS (0.006769 ± 0.020022), LIML (1.041803 ± 4.260779), and FUL (0.052532 ± 0.069872) estimators are positive, which is not expected. This is because these methods are not robust in the presence of invalid instruments. LIML has a higher standard error than other methods because it suffers from a “moments problem,” as noted by Hahn et al.^19^ MR analysis assumes homoscedasticity. In practice, this assumption is often not fulfilled, leading to heteroscedasticity. Additionally, the association between SNPs and the exposure variable is often weak. Therefore, we need to address the issues of many weak instruments and heteroscedasticity. The Sargan test rejects the hypothesis that all the IVs (SNPs) are valid (p-value < 0.001). We use the studentized Breusch–Pagan (BP) test to detect heteroscedasticity in the MR analysis. The results of the BP test show that there is strong evidence of heteroscedasticity (p-value < 0.01). The result of *F*-test = 0.4489 indicates that the SNPs are weakly associated^3^ and Burgess et al.^18^ with exposure variable.

**Table 5.** Estimation results of the causal model with SNPs as Instruments for BMI.

Estimators	$\hat{\beta }$	SE( $\hat{\beta }$ )	95% CI	# Invalid IVs
TSLS	0.006769	0.020022	[−0.03250, 0.04604]	*–*
LIML	1.041803	4.260779	[−7.31474, 9.39835]	*–*
FULL	0.052532	0.069872	[−0.08451, 0.18957]	*–*
PTSLS	−0.008288	0.02150	[−0.05045, 0.03387]	rs1435252, rs6314, rs2241766, rs821616, rs8039957, rs1799945
PLIML	−0.007377	0.00108	[−0.00950, −0.00525]	rs1435252, rs6314, rs2241766, rs8039957
PFUL	−0.007375	0.00107	[−0.00948, −0.00527]	rs1435252, rs6314, rs2241766, rs8039957
PJTSLS	−0.007369	0.01214	[−0.03117, 0.01644]	rs6314, rs2241766, rs8039957
PJLIML	−0.007373	0.00108	[−0.00950, −0.00524]	rs6314, rs2241766, rs8039957
PJFUL	−0.007358	0.00106	[−0.00948, −0.00523]	rs2241766, rs8039957

Note: TSLS = “two-stage least square”; LIML = “limited information maximum likelihood”; FUL = “FUL^17^”; PTSLS = “Penalized TSLS”^12^; proposed estimators: PLIML = “Penalized LIML”; PFUL = “Penalized FUL”; PJTSLS = “Penalized jackknife TSLS”; PJLIML = “Penalized jackknife-LIML”; PJFUL = “Penalized jackknife-FUL”. $\hat{\beta }$ is the estimated coefficient. **^†^** Standard error (SE) and confidence interval (CI) for PTSLS, PLIML, PFUL, PJTSLS, PJLIML, and PJFUL are obtained by bootstrapping. SNP = “single nucleotide polymorphism” (IVs)). “–” means that the TSLS, LIML, and FUL methods do not have the ability to identify any instruments as invalid. These methods are performed under the assumption that all the instruments are valid.

All of the regression coefficients for LJIVE and PKCIV estimation methods are negative, as expected, since these methods are robust with invalid instruments compared to naive *k*-class IV methods. When we use the PKCIV methods, certain instruments are identified as invalid and possibly have direct impacts on HRQLI. In particular, PTSLS (−0.008288 ± 0.02150) identified many instruments as invalid, aligning with the findings of Windmeijer et al.^13^ Furthermore, PLIML (−0.007377 ± 0.00108) and PFUL (−0.007375 ± 0.00107) select the rs1435252, rs6314, rs2241766 and rs8039957 instruments as invalid, all of which could be related to HRQLI. In addition, PJTSLS (−0.007369 ± 0.01214) and PJLIML (−0.007373 ± 0.00108) selects three instruments as invalid while PJFUL (−0.007358 ± 0.00106) selects two instruments as invalid.

We have signs of heteroscedasticity and weak instruments as shown by the BP test and *F*-test. In this situation, the jackknife-based methods are superior according to the simulation results, particularly the PJLIML and PJFUL methods. These methods yield a lower standard error than the naive methods and the PTSLS method proposed by Kang et al.^12^ Further, in contrast to the naive methods, BMI has a negative effect on HRQLI which is the expected sign. One limitation of this analysis is the distribution of the outcome variable. The value of HRQLI ranges from −0.13 to 1.00. A negative HRQLI value represents states that are considered worse than death.^41^ When the data is skewed, one can use the generalized linear model, and if most of the observations are zero, zero-inflated models can be used. HRQLI is unlikely to be normally distributed. If it is constrained to lie between 0 and 1, beta regression can be used. If most of the observations are within the closed unit interval [0, 1], zero/one inflated beta regression could be employed to estimate the causal effects. This approach can extend MR analysis within the generalized linear model framework.

## 6. Concluding remarks

In this paper, a causal model with many weak instruments is examined, where some instruments may directly impact the response variable. We also consider a scenario that includes many instruments with heteroscedastic data. In both of these situations, classic estimators such as NTSLS, NLIML, and NFUL are found to be inconsistent. While the PTSLS estimator is a robust alternative to TSLS in the presence of potentially invalid instruments, its performance may be inadequate when facing many weak instruments, as TSLS estimates are biased toward the probability limit of least square estimates. This bias increases as the degree of overidentification increases.^7^ In this paper, five new methods, PLIML, PFUL, JPTSLS, JPLIML, and JPFUL, are proposed as alternatives to PTSLS for estimating causal effects. The first two estimators, PLIML and PFUL, are extensions of the PTSLS framework. The other three estimators are proposed by using a “leave-one-unit” jackknife-type fitted value in place of the typical first-stage equation. Our empirical findings show that in the presence of weak instruments and heteroscedastic data, both PJLIML and PJFUL outperform PTSLS. When the instruments are not weak, PJTSLS outperforms all the other estimators. Both the simulation results and real-life application results demonstrate that the proposed estimators are robust in estimating IV models with potentially invalid instruments.

The inconsistency of PTSLS, as discussed by Windmeijer et al.,^13^ is that PTSLS may not consistently select invalid instruments if they are relatively strong. This is one of the limitations of the PKCIV methodology. A possible extension of the PKCIV methods is to use the ALASSO procedure and derive the oracle properties. It is a common assumption in IV methods that the instruments are not linearly correlated. However, in practice, genetic variables can be highly correlated, causing the matrix $Z^{T}Z$ to be ill-conditioned, a problem known as multicollinearity. One solution is to use Burgess et al.^38^ methods with principal component analysis to address the issue of correlated variants. Another potential solution could be the application of Tikhonov regularization techniques. Future works could also focus on generalizing the model explored in this paper. Specifically, this led to the consideration of binary exposure variables and nonlinear outcome models, which can be direct extensions of this study. Burgess et al.^54^ introduced an averaging estimator that provides consistent estimates. Furthermore, it would be important to derive the asymptotic distribution and establish the statistical properties for testing hypotheses of the *K*-class and jackknife IVs via the LASSO procedure. Chao et al.^55^ developed the asymptotic distribution of jackknife IV estimators for the classical linear IV model, which could serve as a basis for such extensions.

## Supplemental Material

sj-pdf-1-smm-10.1177_09622802241281035 - Supplemental material for LASSO-type instrumental variable selection methods with an application to Mendelian randomization Supplemental material, sj-pdf-1-smm-10.1177_09622802241281035 for LASSO-type instrumental variable selection methods with an application to Mendelian randomization by Muhammad Qasim, Kristofer Månsson and Narayanaswamy Balakrishnan in Statistical Methods in Medical Research

## Notes

1. The R package is called “pive” and it is available at https://github.com/Qasim-stat/pive.

2.
$e(\hat{\beta },{\hat{\beta }}_{\text{PTSLS}})=median[{\left(\hat{\beta }-\beta_{0}\right)}^{T}(\hat{\beta }-\beta_{0})]/median[{\left({\hat{\beta }}_{\text{PTSLS}}-\beta_{0}\right)}^{T}({\hat{\beta }}_{\text{PTSLS}}-\beta_{0})]$ .

3. “The OLIML is the oracle-LIML estimator that accurately incorporates the invalid instruments (r) as independent variables in the model of interest.”

4. “The NFUL results for the naive-FUL estimator that considers all the instruments as valid when a few of instruments are actually invalid.”

5. Wisconsin Longitudinal Study (WLS): 1957-2020 Version 14.01. Hauser, Robert M., William H. Sewell, and Herd, Pamela. Madison, WI: University of Wisconsin-Madison, WLS; https://researchers.wls.wisc.edu/documentation/

6. We have also analyzed a relatively larger set of IVs (following the exclusion of missing observations, our analysis then included 47 SNPs). The results of the causal effect using a relatively larger set of potential instruments is qualitatively the same to that with 10 SNPs. Results are available from the authors upon request.
