# Supplementary material for: LASSO-type instrumental variable selection methods with an application to Mendelian randomization
Source: Stat Methods Med Res. 2024 Nov 15;34(2):201–23. doi: 10.1177/09622802241281035 (PMC11874601; doi:10.1177/09622802241281035)
Supplement: sj-pdf-1-smm-10.1177_09622802241281035 - Supplemental material for LASSO-type instrumental variable selection methods with an application to Mendelian randomization [file sj-pdf-1-smm-10.1177_09622802241281035.pdf]

Supplement to *“LASSO-type instrumental variable selection methods with an application to Mendelian randomization”*

Muhammad Qasim[a], Kristofer Månsson[a,1] and Narayanaswamy Balakrishnan[b]

[a] Department of economics, finance and statistics, Jönköping University, Jönköping, Sweden
[b] Department of Mathematics and Statistics, McMaster University, Hamilton, Ontario, Canada

Summary

In this supplementary material, we provide additional results from Section 3, along with the proofs for Theorem 3.1 and Lemma 3.3. We also provide additional information on the use of R statistical software[2] to facilitate the Mendelian randomization analysis conducted in the empirical study.

[1] Jönköping International Business School, Gjuterigatan 5, 553 18 Jönköping, Sweden.
Email: kristofer.mansson@ju.se

[2] http://www.r-project.org/

Appendix A

We start by introducing some terminology. Let $\widehat{\boldsymbol{X}} = \mathbb{P}_{\mathrm{Z}}\boldsymbol{X}$, $\mathbb{P}_{\mathrm{Z}} + \mathbb{M}_{\mathrm{Z}} = I_n$, $\mathbb{P}_{\hat{X}} = \mathbb{P}_{\mathrm{Z}}\boldsymbol{X}(\boldsymbol{X}^T\mathbb{P}_{\mathrm{Z}}\boldsymbol{Z})^{-1}\boldsymbol{X}^T\mathbb{P}_{\mathrm{Z}}$, $\mathbb{M}_{\hat{X}} = \boldsymbol{I}_n - \mathbb{P}_{\hat{X}}$, and $\mathbb{P}_{\hat{X}}\mathbb{P}_{\mathrm{Z}} = \mathbb{P}_{\hat{X}}$. Then, the PKCIV estimator can be viewed as

$$\begin{aligned}
\hat{\beta}^{(\lambda)}, \widehat{\boldsymbol{\delta}}^{(\lambda)} &= \underset{\beta,\boldsymbol{\delta}}{\operatorname{argmin}} \frac{1}{2}\|(\boldsymbol{I}_n - k\mathbb{M}_{\mathrm{Z}})(\boldsymbol{Y} - \boldsymbol{X}\beta - \boldsymbol{Z}\boldsymbol{\delta})\|_2^2 + \lambda\|\boldsymbol{\delta}\|_1 \\
&= \underset{\beta,\boldsymbol{\delta}}{\operatorname{argmin}} \frac{1}{2}\|(\mathbb{P}_{\mathrm{Z}} + (1-k)\mathbb{M}_{\mathrm{Z}})(\boldsymbol{Y} - \boldsymbol{X}\beta - \boldsymbol{Z}\boldsymbol{\delta})\|_2^2 + \lambda\|\boldsymbol{\delta}\|_1 \\
&= \underset{\beta,\boldsymbol{\delta}}{\operatorname{argmin}} \frac{1}{2}\|\mathbb{P}_{\mathrm{Z}}(\boldsymbol{Y} - \boldsymbol{X}\beta - \boldsymbol{Z}\boldsymbol{\delta})\|_2^2 + \frac{1}{2}\|(1-k)\mathbb{M}_{\mathrm{Z}}(\boldsymbol{Y} - \boldsymbol{X}\beta - \boldsymbol{Z}\boldsymbol{\delta})\|_2^2 \\
&\quad + \lambda\|\boldsymbol{\delta}\|_1 \\
&= \underset{\beta,\boldsymbol{\delta}}{\operatorname{argmin}} \frac{1}{2}\|(\mathbb{P}_{\hat{X}} + \mathbb{M}_{\hat{X}})\mathbb{P}_{\mathrm{Z}}(\boldsymbol{Y} - \boldsymbol{X}\beta - \boldsymbol{Z}\boldsymbol{\delta})\|_2^2 \\
&\quad + \frac{1}{2}\|(1-k)\mathbb{M}_{\mathrm{Z}}\boldsymbol{Y} - (1-k)\mathbb{M}_{\mathrm{Z}}\boldsymbol{X}\beta\|_2^2 + \lambda\|\boldsymbol{\delta}\|_1 \\
&= \underset{\beta,\boldsymbol{\delta}}{\operatorname{argmin}} \frac{1}{2}\| \mathbb{P}_{\hat{X}}\mathbb{P}_{\mathrm{Z}}(\boldsymbol{Y} - \boldsymbol{X}\beta - \boldsymbol{Z}\boldsymbol{\delta}) \|_2^2 + \frac{1}{2}\| \mathbb{M}_{\hat{X}}\mathbb{P}_{\mathrm{Z}}(\boldsymbol{Y} - \boldsymbol{X}\beta - \boldsymbol{Z}\boldsymbol{\delta}) \|_2^2 \\
&\quad + \frac{1}{2}\| (1-k)\mathbb{M}_{\mathrm{Z}}(\boldsymbol{Y} - \boldsymbol{X}\beta) \|_2^2 + \lambda\|\boldsymbol{\delta}\|_1 \\
&= \underset{\beta,\boldsymbol{\delta}}{\operatorname{argmin}} \frac{1}{2}\left\|\mathbb{P}_{\hat{X}}\left(\boldsymbol{Y} - \boldsymbol{Z}\widehat{\boldsymbol{\delta}}^{(\lambda)}\right) - \widehat{\boldsymbol{X}}\beta\right\|_2^2 + \frac{1}{2}\|\mathbb{M}_{\hat{X}}\mathbb{P}_{\mathrm{Z}}(\boldsymbol{Y} - \boldsymbol{Z}\boldsymbol{\delta})\|_2^2 \\
&\quad + \frac{1}{2}\|(1-\hat{\kappa})\mathbb{M}_{\mathrm{Z}}(\boldsymbol{Y} - \boldsymbol{X}\beta)\|_2^2 + \lambda\|\boldsymbol{\delta}\|_1. \qquad \text{(A1)}
\end{aligned}$$

For $\boldsymbol{\delta} \in \mathbb{R}^L$, the expression $\frac{1}{2}\left\|\mathbb{P}_{\hat{X}}\left(\boldsymbol{Y} - \boldsymbol{Z}\widehat{\boldsymbol{\delta}}^{(\lambda)}\right) - \widehat{\boldsymbol{X}}\beta\right\|_2^2$ in (A1) becomes zero since $\mathbb{P}_{\hat{X}}(\boldsymbol{Y} - \boldsymbol{Z}\boldsymbol{\delta})$ lies within the span of $\widehat{\boldsymbol{X}}$, so we can pick β such that the first expression is zero and the third term in (A1), $\frac{1}{2}\| (1-\hat{\kappa})\mathbb{M}_{\mathrm{Z}}(\boldsymbol{Y} - \boldsymbol{X}\beta) \|_2^2$ is also zero when we take the derivative with respect to $\boldsymbol{\delta}$. Thus, $\widehat{\boldsymbol{\delta}}^{(\lambda)}$ for $\lambda > 0$ can be found as

$$\widehat{\boldsymbol{\delta}}^{(\lambda)} = \underset{\boldsymbol{\delta}}{\operatorname{argmin}} \frac{1}{2}\left\|\widetilde{\boldsymbol{Y}} - \widetilde{\boldsymbol{Z}}\boldsymbol{\delta}\right\|_2^2 + \lambda\|\boldsymbol{\delta}\|_1, \qquad \text{(A2)}$$

where $\widetilde{\boldsymbol{Y}} = \mathbb{M}_{\hat{X}}\mathbb{P}_{\mathrm{Z}}\boldsymbol{Y}$ and $\widetilde{\boldsymbol{Z}} = \mathbb{M}_{\hat{X}}\boldsymbol{Z}$. The estimator in (A2) seems to be a classic LASSO estimator with outcome variables $\mathbb{M}_{\hat{X}}\mathbb{P}_{\mathrm{Z}}\boldsymbol{Y}$ and $\mathbb{M}_{\hat{X}}\boldsymbol{Z}$ as explanatory variables. Given the estimator in (A2), $\widehat{\boldsymbol{\delta}}^{(\lambda)}$, we minimize (A1) with respect to β to estimate the causal effect as

$$\hat{\beta}^{(\lambda)} = \underset{\beta}{\operatorname{argmin}} \frac{1}{2} \left\| \mathbb{P}_{\hat{X}}\left(\boldsymbol{Y} - \boldsymbol{Z}\hat{\boldsymbol{\delta}}^{(\lambda)}\right) - \hat{\boldsymbol{X}}\beta \right\|_2^2 + \frac{1}{2} \|(1-\hat{\kappa})\mathbb{M}_{Z}(\boldsymbol{Y} - \boldsymbol{X}\beta)\|_2^2.$$

Appendix B

Additional results from Section 3

Lemma 3.1: Let $\mathbb{A}$ be any matrix and let q and q' be any positive integers. Then, we have

$$2\left[\theta_{q,q'}(\mathbb{A})\right] \leq \Delta_{q+q'}^{+}(\mathbb{A}) - \Delta_{q+q'}^{-}(\mathbb{A}). \quad \text{(B1)}$$

Lemma 3.1 is given in Cai et al. (2010) and Kang et al. (2016), and it connects the RI property and ROC conditions.

We next illustrate the use of l_1-regularization with sparsity to solve the signal denoising, compression, and approximation problems. We first redefine the standard properties of the conventional LASSO method by considering the traditional linear model

$$Y_i = \boldsymbol{Z}_{i.}^T \boldsymbol{\delta}_0 + e_i, \quad \text{(B2)}$$

where $\boldsymbol{Z} \in \mathbb{R}^{n \times p}$ has lower and upper RI constants $\Delta_r^{-}(\boldsymbol{Z})$ and $\Delta_r^{+}(\boldsymbol{Z})$, respectively, vector $\boldsymbol{\delta} \in \mathbb{R}^p$ r-sparse and $r \in \{1,2,\ldots,p\}$. In addition, suppose that $\boldsymbol{\delta}_{\max(r)}$ are the r-largest entries of $\boldsymbol{\delta}$, meaning that the vector $\boldsymbol{\delta}$ has all but the r-largest entries as zero, and $\boldsymbol{\delta}_{\max(r)}^{-} = \boldsymbol{\delta} - \boldsymbol{\delta}_{\max(r)}$, which is the basic setting in the compressed sensing literature (see Cai et al., 2010 and Cai and Zhang, 2013). The LASSO estimator is then

$$\hat{\boldsymbol{\delta}} = \underset{\boldsymbol{\delta} \in \mathbb{R}^p}{\operatorname{argmin}} \frac{1}{2} \| Y - \boldsymbol{Z}\boldsymbol{\delta} \|_2^2 + \lambda \| \boldsymbol{\delta} \|_1. \quad \text{(B3)}$$

Lemma 3.2: Suppose that the model matrix $\boldsymbol{Z}$ in (B2) has lower and upper restricted isometry properties with a parameter vector $\boldsymbol{\delta}$ that is r-sparse.

i. *If $\lambda \geq \tau \| \boldsymbol{Z}^T \boldsymbol{e} \|_\infty$ and $\boldsymbol{e} = Y - \boldsymbol{Z}\boldsymbol{\delta}$ for some $\tau > 1$, then any estimate based on the LASSO (B3) satisfies the bound*

$$\| \left(\hat{\boldsymbol{\delta}} - \boldsymbol{\delta}_0\right)_{\max(r)}^{-} \|_1 \leq \left(\frac{\tau+1}{\tau-1}\right) \| \left(\hat{\boldsymbol{\delta}} - \boldsymbol{\delta}_0\right)_{\max(r)} \|_1; \quad \text{(B4)}$$

ii. *Given the condition $\Delta_{2\tau}^{-}(\boldsymbol{Z}) > \frac{\tau+1}{3\tau-1}[\Delta_{2\tau}^{+}(\boldsymbol{Z})]$, any estimate from the regularized method (B3) satisfies the bound*

$$\| \left(\hat{\boldsymbol{\delta}} - \boldsymbol{\delta}_0\right)_{\max(r)} \|_2 \le \frac{2(\tau^2-1)\lambda}{\tau[(3\tau-1)\Delta_{2\tau}^{-}(\boldsymbol{Z})]-\tau[(\tau+1)\Delta_{2\tau}^{+}(\boldsymbol{Z})]}. \tag{B5}$$

Proof: Proving the inequality in (B4) is straightforward, whereas proving the bound in (B5) requires additional work. Specifically, to prove the bound in (B5), we can utilize the ROC definition in (2.2), along with Lemma 5.1 and Lemma 5.4 from Cai and Zhang (2013), as well as Lemma 3.1 results in (B1). The detailed results of Lemma 3.2 can be found in Kang et al. (2016). □

Proof of Theorem 3.1:

Theorem 3.1 shows the performance of the PKCIV estimators, wherein Lemmas 3.1 and 3.2 are used to prove the result. The estimator $\hat{\beta}^{(\lambda)}$ can be modified as

$$\hat{\beta}^{(\lambda)} = \frac{\widetilde{\boldsymbol{X}}^T\boldsymbol{Y} - \widehat{\boldsymbol{X}}^T\boldsymbol{Z}\hat{\boldsymbol{\delta}}^{(\lambda)}}{\|\widehat{\boldsymbol{X}}\|_2^2 + d\left(\|\boldsymbol{X}\|_2^2 - \|\widehat{\boldsymbol{X}}\|_2^2\right)} = \frac{\widetilde{\boldsymbol{X}}^T(\boldsymbol{X}\beta_0 + \boldsymbol{Z}\boldsymbol{\delta}_0 + \boldsymbol{e}) - \widehat{\boldsymbol{X}}^T\boldsymbol{Z}\hat{\boldsymbol{\delta}}^{(\lambda)}}{\|\widehat{\boldsymbol{X}}\|_2^2 + d\left(\|\boldsymbol{X}\|_2^2 - \|\widehat{\boldsymbol{X}}\|_2^2\right)}$$

Because $\widehat{\boldsymbol{X}}^{\boldsymbol{T}} = \boldsymbol{X}^{\boldsymbol{T}}\mathbb{P}_{\mathrm{Z}}$ and $\mathbb{P}_{\hat{X}}\mathbb{P}_{\mathrm{Z}} = \mathbb{P}_{\hat{X}}$, along with the projection matrix properties, we obtain

$$\hat{\beta}^{(\lambda)} - \beta_0 = \frac{\left(\widehat{\boldsymbol{X}}^T + d\left(\boldsymbol{X}^T - \widehat{\boldsymbol{X}}^T\right)\right)}{\|\widehat{\boldsymbol{X}}\|_2^2 + d\left(\|\boldsymbol{X}\|_2^2 - \|\widehat{\boldsymbol{X}}\|_2^2\right)}\boldsymbol{e} - \frac{\mathbb{P}_{\hat{X}}\boldsymbol{Z}}{\|\widehat{\boldsymbol{X}}\|_2 + d\left(\|\boldsymbol{X}\|_2 - \|\widehat{\boldsymbol{X}}\|_2\right)}\left(\hat{\boldsymbol{\delta}}^{(\lambda)} - \boldsymbol{\delta}_0\right).$$

The second part of the theorem takes the norm on both sides of the above equation, yielding the inequality

$$\left\|\hat{\beta}^{(\lambda)} - \beta_0\right\|_2 \le \frac{\left\|\mathbb{P}_{\hat{X}}\boldsymbol{Z}\left(\hat{\boldsymbol{\delta}}^{(\lambda)} - \boldsymbol{\delta}_0\right)\right\|_2}{\|\widehat{\boldsymbol{X}}\|_2 + d\left(\|\boldsymbol{X}\|_2 - \|\widehat{\boldsymbol{X}}\|_2\right)} + \frac{\left|\left(\widehat{\boldsymbol{X}}^{\boldsymbol{T}} + d\left(\boldsymbol{X}^{\boldsymbol{T}} - \widehat{\boldsymbol{X}}^{\boldsymbol{T}}\right)\right)\boldsymbol{e}\right|}{\|\widehat{\boldsymbol{X}}\|_2^2 + d\left(\|\boldsymbol{X}\|_2^2 - \|\widehat{\boldsymbol{X}}\|_2^2\right)}. \tag{B6}$$

The bound on $\left\|\mathbb{P}_{\hat{X}}\boldsymbol{Z}\left(\hat{\boldsymbol{\delta}}^{(\lambda)} - \boldsymbol{\delta}_0\right)\right\|_2$ remains essential to bound $\left\|\hat{\beta}^{(\lambda)} - \beta_0\right\|_2$. We first prove the inequality on $\left\|\mathbb{P}_{\hat{X}}\boldsymbol{Z}\left(\hat{\boldsymbol{\delta}}^{(\lambda)} - \boldsymbol{\delta}_0\right)\right\|_2$. For this, we have to convert the bound for $\left\|\left(\hat{\boldsymbol{\delta}} - \boldsymbol{\delta}_0\right)_{\max(r)}\right\|_2$ in part (ii) of Lemma 3.2 to the bound for $\left\|\mathbb{P}_{\hat{X}}\boldsymbol{Z}\left(\hat{\boldsymbol{\delta}}^{(\lambda)} - \boldsymbol{\delta}_0\right)\right\|_2$, where r is the number of invalid instruments. We set the RI property constants to exploit Lemma 3.2. By assumption, we know that $3\|\boldsymbol{Z}^T\mathbb{M}_{\hat{X}}\mathbb{P}_{\mathrm{Z}}\boldsymbol{e}\|_\infty \le \lambda$ if Model (2.1) is modified as $\mathbb{M}_{\hat{X}}\mathbb{P}_{\mathrm{Z}}\boldsymbol{Y} = \mathbb{M}_{\hat{X}}\mathbb{P}_{\mathrm{Z}}\boldsymbol{Z}\boldsymbol{\delta}_0 + \mathbb{M}_{\hat{X}}\mathbb{P}_{\mathrm{Z}}\boldsymbol{e}$ in step I of the algorithm. Thus, we link the RI constants of $\mathbb{M}_{\hat{X}}\boldsymbol{Z}$ with the matrix $\boldsymbol{Z}$ (Kang et al., 2016) for any $2r$-sparse vector $x \in \mathbb{R}^L$. This suggests that

$$\|(\boldsymbol{I}_n - \mathbb{P}_{\hat{X}})\boldsymbol{Z}x\|_2^2 \; = \; \|\boldsymbol{Z}x\|_2^2 - \|\mathbb{P}_{\hat{X}}\boldsymbol{Z}x\|_2^2 \leq \|\boldsymbol{Z}x\|_2^2 \leq \Delta_{2r}^{+}(\boldsymbol{Z})\|x\|_2^2 \tag{B7}$$

and

$$\|(\boldsymbol{I}_n - \mathbb{P}_{\hat{X}})\boldsymbol{Z}x\|_2^2 \geq \Delta_{2r}^{-}(\boldsymbol{Z})\|x\|_2^2 - \Delta_{2r}^{+}(\mathbb{P}_{\hat{X}}\boldsymbol{Z})\|x\|_2^2. \tag{B8}$$

Hence, by using (B7) and (B8), we obtain the following RI constant constraints:

$$\Delta_{2r}^{+}(\mathbb{M}_{\hat{X}}\boldsymbol{Z}) \leq \Delta_{2r}^{+}(\boldsymbol{Z}), \tag{B9}$$

$$\Delta_{2r}^{-}(\mathbb{M}_{\hat{X}}\boldsymbol{Z}) \geq \Delta_{2r}^{-}(\boldsymbol{Z}) - \Delta_{2r}^{+}(\mathbb{P}_{\hat{X}}\boldsymbol{Z}). \tag{B10}$$

We now use Lemma 3.2 and inequalities (B9) and (B10) with the condition that $2\big(\Delta_{2r}^{-}(\boldsymbol{Z}) - \Delta_{2r}^{+}(\mathbb{P}_{\hat{X}}\boldsymbol{Z})\big) > \Delta_{2r}^{+}(\boldsymbol{Z})$ to obtain

$$\left\|\big(\widehat{\boldsymbol{\delta}} - \boldsymbol{\delta}_0\big)_{\max(r)}\right\|_2 \leq \left(\frac{2(\tau^2 - 1)\lambda/\tau}{(3\tau - 1)\Delta_{2r}^{-}(\mathbb{M}_{\hat{X}}\boldsymbol{Z}) - (\tau + 1)\Delta_{2r}^{+}(\mathbb{M}_{\hat{X}}\boldsymbol{Z})}\right),$$

We now set $\tau = 3$ by the assumption $\{\boldsymbol{e} \in \mathbb{R}^n\colon \|\boldsymbol{Z}^T\mathbb{M}_{\hat{X}}\mathbb{P}_{\mathrm{Z}}\boldsymbol{e}\|_\infty \leq \lambda/3\}$ and link the restricted isometry property constants of $\Delta_{2r}^{+}(\mathbb{M}_{\hat{X}}\boldsymbol{Z})$ and $\Delta_{2r}^{-}(\mathbb{M}_{\hat{X}}\boldsymbol{Z})$ to obtain

$$\left\|\big(\widehat{\boldsymbol{\delta}} - \boldsymbol{\delta}_0\big)_{\max(r)}\right\|_2 \leq \frac{4\lambda}{6\big(\Delta_{2r}^{-}(\boldsymbol{Z}) - \Delta_{2r}^{+}(\mathbb{P}_{\hat{X}}\boldsymbol{Z})\big) - 3\Delta_{2r}^{+}(\boldsymbol{Z})} \tag{B11}$$

and

$$\left\|\big(\widehat{\boldsymbol{\delta}} - \boldsymbol{\delta}_0\big)_{\max(r)}\right\|_2 \leq 2\left\|\big(\widehat{\boldsymbol{\delta}} - \boldsymbol{\delta}_0\big)_{\max(r)}\right\|_1.$$

Next, we use the results of Lemma 1.1 (Cai and Zhang, 2014) and Theorem 2 (Kang et al., 2016) to show that $\big(\widehat{\boldsymbol{\delta}} - \boldsymbol{\delta}_0\big)_{\max(r)}^{-}$ is a weighted average of r-sparse vectors. Hence,

$$\widehat{\boldsymbol{\delta}} - \boldsymbol{\delta}_0 = \sum_{i=1}^{n} \lambda_i\left(\big(\widehat{\boldsymbol{\delta}} - \boldsymbol{\delta}_0\big)_{\max(r)} + \nu_i\right),$$

where $\nu_i \in \mathbb{R}^L$, $\operatorname{supp}(\nu_i) \subseteq \operatorname{supp}\left(\big(\widehat{\boldsymbol{\delta}} - \boldsymbol{\delta}_0\big)_{\max(r)}^{-}\right)$, $\|\nu_i\|_1 \leq \left\|\big(\widehat{\boldsymbol{\delta}} - \boldsymbol{\delta}_0\big)_{\max(r)}\right\|_1$, $\|\nu_i\|_\infty \leq \frac{2}{r}\left\|\big(\widehat{\boldsymbol{\delta}} - \boldsymbol{\delta}_0\big)_{\max(r)}\right\|_1$ and $\left\|\big(\widehat{\boldsymbol{\delta}} - \boldsymbol{\delta}_0\big)_{\max(r)} + \nu_i\right\|_2^2 = \left\|\big(\widehat{\boldsymbol{\delta}} - \boldsymbol{\delta}_0\big)_{\max(r)}\right\|_2^2 +$

$\|\nu_i\|_2^2 \leq 5\left\|\left(\widehat{\boldsymbol{\delta}}-\boldsymbol{\delta}_0\right)_{\max(r)}\right\|_2^2$. Then, the bound for $\left\|\mathbb{P}_{\hat{X}}\boldsymbol{Z}\left(\widehat{\boldsymbol{\delta}}^{(\lambda)}-\boldsymbol{\delta}_0\right)\right\|_{\mathbf{2}}$, which is based on the inequality given in (B11), is

$$\begin{aligned}
&\left\|\mathbb{P}_{\hat{X}}\boldsymbol{Z}\left(\widehat{\boldsymbol{\delta}}^{(\lambda)}-\boldsymbol{\delta}_0\right)\right\|_{\mathbf{2}} \\
&\qquad \leq \sum_{i=1}^{N}\lambda_i\left\|\mathbb{P}_{\hat{X}}\boldsymbol{Z}\left(\widehat{\boldsymbol{\delta}}-\boldsymbol{\delta}_0\right)_{\max(r)}+\nu_i\right\|_2^2 \\
&\qquad \leq \sum_{i=1}^{N}\lambda_i\left(5\varDelta_{2r}^{+}(\mathbb{P}_{\hat{X}}\boldsymbol{Z})\right)^{1/2}\left\|\left(\widehat{\boldsymbol{\delta}}-\boldsymbol{\delta}_0\right)_{\max(r)}\right\|_2^2 \\
&\qquad = \frac{4\lambda\left(5\varDelta_{2r}^{+}(\mathbb{P}_{\hat{X}}\boldsymbol{Z})\right)^{1/2}}{6\left(\varDelta_{2r}^{-}(\boldsymbol{Z})-\varDelta_{2r}^{+}(\mathbb{P}_{\hat{X}}\boldsymbol{Z})\right)-3\varDelta_{2r}^{+}(\boldsymbol{Z})}.
\end{aligned}$$

This completes the proof of the second part of the theorem. □

Appendix C

Proof of Lemma 3.3: Suppose that $X_{(-i)}$ is an $(n-1)\times 1$ vector given by $\mathbf{X}$ with the ith row removed; similarly, $\boldsymbol{Z}_{(-i)}$ is an $(n-1)\times L$ matrix with the ith row removed. Then, the estimate of the parameter vector $\boldsymbol{\psi}$ using all observations except observation i is

$$\widehat{\boldsymbol{\psi}}_{(-i)} = \left(\boldsymbol{Z}_{(-i)}^T\boldsymbol{Z}_{(-i)}\right)^{-1}\boldsymbol{Z}_{(-i)}^T X_{(-i)}.$$

The ith row of the optimal instrument can be computed as $\boldsymbol{Z}_{i.}^T\widehat{\boldsymbol{\psi}}_{(-i)}$ rather than $\boldsymbol{Z}_{i.}^T\widehat{\boldsymbol{\psi}}$. Thus, we obtain

$$\begin{aligned}
\mathbb{E}\left[\hat{X}_{(-i)}^T e_i\right] &= \mathbb{E}\left[\hat{\psi}_{(-i)}^T\boldsymbol{Z}_{i.}^T e_i\right] \\
&= \mathbb{E}\left\{\left(X_{(-i)}^T\boldsymbol{Z}_{(-i)}\left(\boldsymbol{Z}_{(-i)}^T\boldsymbol{Z}_{(-i)}\right)^{-1}\boldsymbol{Z}_{i.}^T e_i\right)|\boldsymbol{Z}\right\} \\
&= \mathbb{E}\left[\boldsymbol{Z}_{(-i)}\left(\boldsymbol{Z}_{(-i)}^T\boldsymbol{Z}_{(-i)}\right)^{-1}\boldsymbol{Z}_{i.}^T\mathbb{E}\left\{X_{(-i)}^T e_i|\boldsymbol{Z}\right\}\right] = 0.
\end{aligned}$$

Hence, $\mathbb{E}\left[\hat{X}_{(-i)}^T e_i\right] = 0$ since X_j is independent of e_i if $j \neq i$, as needed. □

Software

We introduce a function called "pive" that is coded in the R statistical software and can be employed to apply the proposed methods. The performance of these proposed approaches is compared with the existing methods, specifically two-stage least squares (TSLS), limited information maximum likelihood (LIML), FUL (Fuller, 1977), and penalized TSLS (PTSLS). Below, we provide information on the implementation of proposed and existing methods using R software:

1. TSLS, LIML, and FUL are computed applying the "ivmodel" package (Kang et al., 2021).
2. The "sisVIVE" R package (Kang 2017) is used for PTSLS, which is a special form of penalized *K*-class instrumental variable (IV) estimator (PKCIVE), and can be estimated with our R package "pive". This package "pive" is used for all the proposed methods. More information about the R implementation is available on GitHub [https://github.com/Qasim-stat/pive].

The R code for Mendelian randomization analysis is as follows:

```
# Install and load the necessary packages ----
rm(list=ls()) ##Clear the memory
install.packages("ivmodel")
install.packages("sisVIVE")
devtools::install_github("Qasim-stat/pive")
library(ivmodel) #Instrumental variables modeling
library(sisVIVE)   #Package: sisVIVE (Kang et al., 2016)
library(pive)      #Package: pive for proposed methods
library(MASS)      #Statistical analysis tools
library(readxl)    #Read Excel files
library(openxlsx) #Save the excel output


mr_df <- read_excel("mr_dataset.xlsx")
```

```
n <- nrow(Z); L <- ncol(Z)

## TSLS, LIML, and FUL Estimation from the 'ivmodel' Package ----

fit_ivmodel <- ivmodel(Y = Y, D = X, Z = Z, k = 1)
beta_tsls <- c(fit_ivmodel$kClass$point.est)
beta_liml <- c(fit_ivmodel$LIML$point.est)
beta_ful  <- c(fit_ivmodel$Fuller$point.est)
kliml <- fit_ivmodel$LIML$k
kful  <- fit_ivmodel$Fuller$k

## Save the SE and compute 95% CI for the estimates ----
se_beta_tsls <- c(fit_ivmodel$kClass$std.err)
se_beta_liml <- c(fit_ivmodel$LIML$std.err)
se_beta_ful  <- c(fit_ivmodel$Fuller$std.err)

ci_tsls_lower <- beta_tsls - 1.96 * se_beta_tsls
ci_tsls_upper <- beta_tsls + 1.96 * se_beta_tsls
ci_liml_lower <- beta_liml - 1.96 * se_beta_liml
ci_liml_upper <- beta_liml + 1.96 * se_beta_liml
ci_ful_lower <- beta_ful - 1.96 * se_beta_ful
ci_ful_upper <- beta_ful + 1.96 * se_beta_ful

# Lasso-type IV methods ----
  fit_pivmodel <- pive(Y,X,Z,bootstrap = FALSE)

  beta_ptsls  <- fit_pivmodel$Estimate[1]; #cv.sisVIVE(Y,X,Z)
  beta_pliml  <- fit_pivmodel$Estimate[2]; #cv.PKCIVE(Y,X,Z,k = kliml)
  beta_pful   <- fit_pivmodel$Estimate[3]; #cv.PKCIVE(Y,X,Z,k = kful)
  beta_pjtsls <- fit_pivmodel$Estimate[4]; #cv.LJIVE (Y,X,Z,k = 1)
  beta_pjliml <- fit_pivmodel$Estimate[5]; #cv.LJIVE (Y,X,Z,k = klimlj)
```

```
  beta_pjful  <- fit_pivmodel$Estimate[6]; #cv.LJIVE (Y,X,Z,k = kfulj)



  # Combine all coefficients ----
  coefs <- cbind(beta_tsls, beta_liml, beta_ful, beta_ptsls, beta_pliml
           , beta_pful, beta_pjtsls, beta_pjliml,beta_pjful)
  colnames(coefs)                    <-                                   c("TSLS",
"LIML","FUL","PTSLS","PLIML","PFUL","JPTSLS","JPLIML","JPFUL")

# Perform bootstrap to calculate the SE and 95% CI for penalized IV methods ----

boot_results <- pive(Y,X,Z,bootstrap = TRUE, B = 500, alpha = 0.05)
boot_se      <- cbind(boot_results$Std.error)
boot_ci_lower<- cbind(boot_results$`CI Lower`)
boot_ci_upper<- cbind(boot_results$`CI Upper`)

# Create results data frame ----
se   <- rbind(se_beta_tsls,se_beta_liml,se_beta_ful,boot_se)
cill <- rbind(ci_tsls_lower,ci_liml_lower,ci_ful_lower,boot_ci_lower)
ciup <- rbind(ci_tsls_upper,ci_liml_upper,ci_ful_upper,boot_ci_upper)

results <- data.frame(
  Coefficient  = t(coefs),
  `Std.Error` = se,
  `LL%` = cill,
  `UL%` = ciup
)

row.names(results) <- c("TSLS", "LIML","FUL","PTSLS","PLIML","PFUL",
                      "JPTSLS","JPLIML","JPFUL")
```

```
print("Results for all estimators:")
print(results)

# Write the data to the excel sheet ----
OUT <- createWorkbook()
resname <- paste("estimated_results-",".",L,".xlsx",sep="")
addWorksheet(OUT, resname)
writeData(OUT, sheet = resname , x = results, rowNames = TRUE)
saveWorkbook(OUT, resname)
```

References:

Fuller, W. A. (1977). Some properties of a modification of the limited information estimator. Econometrica: *Journal of the Econometric Society*, 939-953.

Kang, H., Jiang, Y., Zhao, Q., & Small, D. S. (2021). Ivmodel: an R package for inference and sensitivity analysis of instrumental variables models with one endogenous variable. *Observational Studies*, 7(2), 1-24.

Kang, H. (2017). sisVIVE: Some invalid some valid instrumental variables estimator. R package version, 1.
